# Grafted Coiled-Coil Peptides as Multivalent Scaffolds for Protein Recognition

**DOI:** 10.1021/acschembio.5c00137

**Published:** 2025-06-05

**Authors:** Amanda M. Acevedo-Jake, Bram Mylemans, Danielle F. Kay, Peiyu Zhang, Boguslawa Korona, Guto G. Rhys, Aneika C. Leney, Danny T. Huang, Thomas A. Edwards, Laura Itzhaki, Derek N. Woolfson, Andrew J. Wilson

**Affiliations:** † School of Chemistry, 1724 University of Birmingham, Edgbaston B15 2TT, U.K.; ‡ School of Chemistry, 1980 University of Bristol, Cantock’s Close, Bristol BS8 1TS, U.K.; § School of Biosciences, 1724 University of Birmingham, Edgbaston B15 2TT, U.K.; ∥ School of Chemistry, 4468 University of Leeds, Woodhouse Lane, Leeds LS2 9JT, U.K.; ⊥ Department of Pharmacology, 2152 University of Cambridge, Cambridge CB2 1PD, U.K.; # School of Chemistry, 2112 Cardiff University, Main Building, Park Place, Cardiff CF10 3AT, U.K.; ∇ 3532 Cancer Research UK Scotland Institute, Garscube Estate, Switchback Road, Glasgow G61 1BD, U.K.; ○ School of Cancer Sciences, University of Glasgow, Garscube Estate, Switchback Road, Glasgow G61 1QH, U.K.; ◆ College of Biomedical Sciences, 422238 Larkin University, 18301 N Miami Avenue #1, Miami, Florida 33169 , United States; ¶ School of Biochemistry, Medical Sciences Building, University of Bristol, University Walk, Bristol BS8 1TD, U.K.; & BrisSynBio, 1980 University of Bristol, Life Sciences Building, Tyndall Avenue, Bristol BS8 1TQ, U.K.

## Abstract

Self-assembled peptides are promising templates for the design of inhibitors of protein–protein interactions (PPIs) because they can be endowed with affinity- and selectivity-defining amino acids alongside favorable physicochemical properties such as solubility and stability. Here, we describe a tunable coiled-coil scaffold and its interaction with MCL-1, an α-helix-binding antiapoptotic protein and important target in oncology. We explore the role of oligomerization, multivalency, and cooperativity in PPI inhibition. Hot-spot residues from an MCL-1 binding peptide (NOXA-B) are grafted onto the outer surfaces of homo- and heterodimeric coiled-coil peptides to obtain inhibitors with mid-nM potency and selectivity over BCL-x_L_. Binding of homodimeric coiled coils to MCL-1 is positively cooperative, resulting in stabilization of both partners. Homodimeric coiled coils support the binding of two copies of the target protein. Modification of the coiled-coil sequence to favor assembly of higher-order scaffolds (trimer and tetramer) negatively impacts inhibitory potency, with AlphaFold2 modeling and biophysical data indicating a complex interplay between coiled-coil oligomerization and target binding. Together, these data establish dimeric coiled coils as the most promising of such scaffolds to develop inhibitors of α-helix-mediated PPIs.

## Graphical abstract

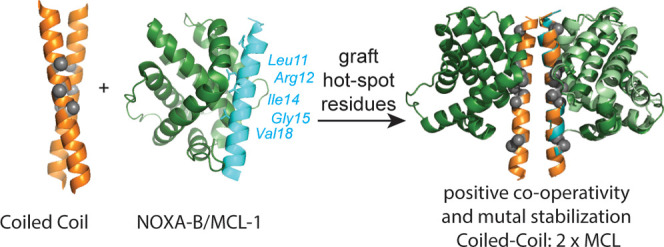

## Introduction

Protein–protein interactions (PPIs) control and regulate many biological processes, including cell signaling, homeostasis, and immune response.^1–3^ Modulation of PPIs offers significant therapeutic potential. Indeed, there has been progress in developing small molecules, antibodies, and peptides for neurodegenerative disorders, inflammatory diseases, and cancer.^4–6^ However, it remains challenging to develop orthosteric inhibitors of PPIs, as recognition typically occurs across a large surface area that often lacks the structurally well-defined pockets that are the hallmark of ligand binding sites. Peptides are a promising class of PPI inhibitors.^6^ They offer extended and structurally diverse three-dimensional surfaces that can be endowed with amino-acid–based recognition handles to confer the necessary target binding affinity and selectivity. Their straightforward synthesis and purification,^7^ potential for chemical modification or stabilization, and crucially, their ability to mimic a native binding partner with high fidelity,^6^ have stimulated interest in their use as PPI inhibitors. Lastly, significant developments in protein design^8–10^ have made possible the design of stable and soluble scaffolds for presenting binding epitopes.^11–20^ Coiled-coil peptide assemblies in particular are inherently stable, highly modular and well-understood scaffolds.^21,22^ Coiled-coil sequences have distinctive (*abcdefg*)_*n*_ heptad repeats where the *a* and *d* positions are usually occupied by hydrophobic residues to promote the association of two or more α-helical peptides.^21^ Such helical bundles can present binding motifs in a spatially defined manner on the outer surfaces of the assemblies. These scaffolds have been exploited to rewire signaling pathways and regulate signaling, in delivery, and as therapeutic candidates.^23–31^ Moreover, coiled-coil oligomers^32,33^ offer a robust platform to achieve multivalent or cooperative binding.^28,34^ Multiple binding sites can improve potency through avidity and induce signaling events by colocalization.^35–37^ Positive cooperative binding may occur where ligand binding increases the affinity of subsequent ligand binding events.^38^

Previously, we described the use of coiled coils as scaffolds for inhibition of helix-mediated PPIs. In that work, clusters of hot-spot residues from NOXA-B are grafted onto parallel homo- and heterodimeric coiled coils, which selectively inhibit the interaction of BH3 ligands with MCL-1.^39^ MCL-1 is a member of the B-cell lymphoma 2 (BCL-2) family of proteins. This family regulates apoptosis through interactions between pro- and antiapoptotic members.^40–42^ Therefore, MCL-1 has been the focus of drug discovery efforts.^43,44^ In our prior work, we did not establish if the coiled coils engage MCL-1 as a dimer or dissociate upon protein binding. Thus, here we explore further coiled-coil scaffolds, including homotrimeric and tetrameric coiled coils alongside a monomeric control peptide to investigate the interplay between MCL-1 inhibitory potency and coiled-coil assembly (Figure 1a,b). Using a combination of AlphaFold2 modeling, fluorescence anisotropy, circular dichroism (CD) spectroscopy, native mass spectrometry, size-exclusion chromatography (SEC), and analytical ultracentrifugation (AUC), we show that the homodimeric coiled coil is the optimal scaffold for these designed inhibitors targeting MCL-1. Binding occurs with 2:2 peptide:protein stoichiometry (i.e., each coiled-coil dimer binds two protein targets), and it is cooperative, leading to mutual stabilization of both the coiled-coil scaffold and the target protein. The ability to recruit a binding partner for each recognition surface of the coiled coil opens the path toward future design of multispecific molecules that can act as “molecular glues” for ternary complex formation.^45^

**Figure 1:**
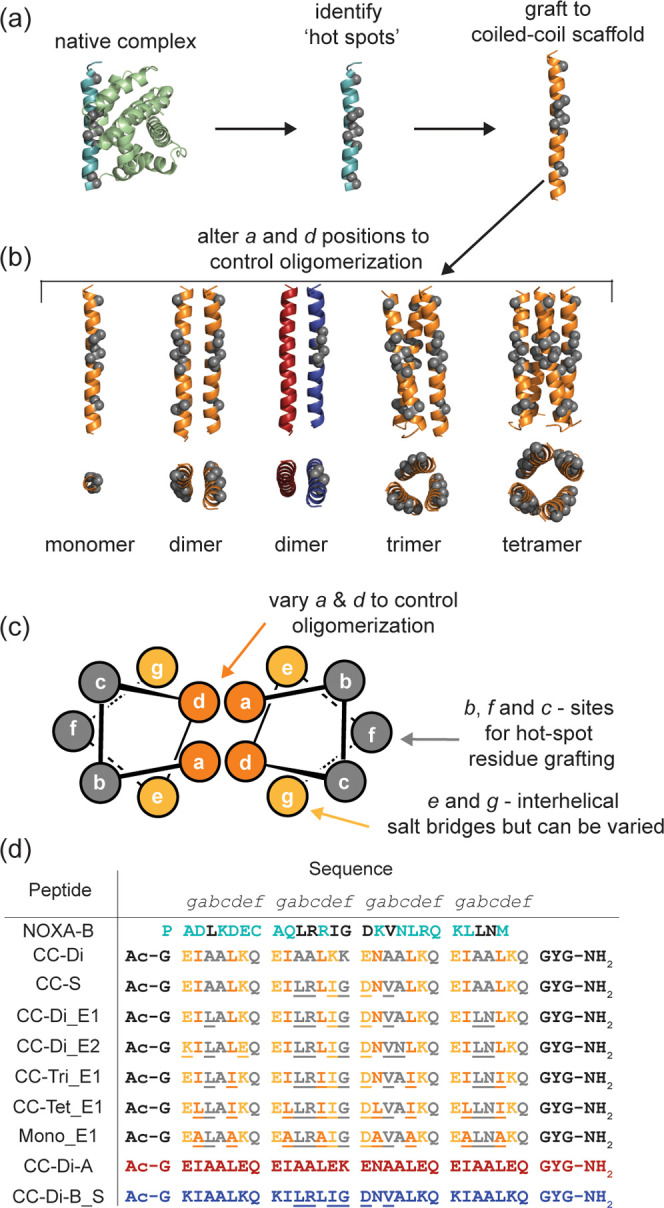
Overview of study: (a) Using the NOXA-B/MCL-1 complex (PDB ID: 2JM6, first model) “hot-spot” residues^39^ (shown as gray spheres) from NOXA-B are identified and grafted onto the outer surface of a coiled-coil sequence; (b) Altering the *a* and *d* positions in the heptad sequence controls the oligomerization state of the coiled-coil assembly,^21,47^ and is used to generate “hot-spot” grafted scaffolds representing a monomeric peptide, and homo- and heterodimeric, homotrimeric, and homotetrameric coiled coils; (c) Helical-wheel diagram depicting a parallel dimeric coiled coil with hydrophobic core residues (dark orange) flanking electrostatic residues (light orange) and variable solvent exposed residues (gray highlighted); (d) sequences and helical wheel alignment of peptides used in this study: NOXA-B (cyan) with amino acids (black) signifying ‘hot-spot’ residues. The canonical parent CC-Di (color coding matching panel (c) is used to generate sequences that assemble into parallel homomeric coiled coils with varied residues underlined) and a parallel heterodimeric coiled coil (acidic partner in red, basic partner in blue, and varied residues underlined).

## Results and Discussion

### Designed Coiled-Coil Scaffolds That Mimic NOXA-B

Coiled Coils are often defined by characteristic 7-residue (heptad) repeats. In a helical conformation, the *a* and *d* positions are typically hydrophobic and mediate assembly through “knob-into-holes” packing. Modification of the *a* and *d* positions in the heptad can be exploited to alter the coiled-coil assembly state.^21,32^ The *e* and *g* residues are frequently complementary charged residues, while the *b*, *c,* and *f* positions can be varied (Figure 1c). Previously, we have identified NOXA-B residues predicted to contribute to MCL-1 binding.^39^ This gives three different binding sequences of varying length: a short motif (S), composed of the centrally clustered residues Leu11, Arg12, Ile14, Asp16 and Val18; a first extended motif (E1), comprising the short motif residues plus Leu4, Leu25 and Asn26; and, a second extended motif (E2) incorporating the E1 residues as well as Glu7, Asn19, and glutamate and lysine at positions 2 and 7 to maintain interchain salt bridges within the coiled coil (Figure 1d). The central Gly15, which contacts the surface of MCL-1 and is highly conserved in BH3 sequences,^46^ is included in all three motifs. The identified binding motifs have been grafted onto the outer face of the well-characterized *de novo* designed peptide CC-Di,^32^ avoiding substitutions at the *a* and *d* heptad positions as these drive coiled-coil assembly.^47^ The parallel homodimeric coiled-coil sequences CC-Di_S, CC-Di_E1, and CC-Di_E2 ^39^ display copies of the grafted motifs S, E1, and E2, on both outer faces of the assembly. A parallel heterodimeric pair comprising an acidic peptide (CC-Di-A) partnered with basic peptide (CC-Di-B_S) bearing the S motif is also described.^39^ These designed coiled coils selectively inhibit the NOXA-B/MCL-1 interaction with a clear positive correlation between potency and the number of hot-spot residues included in the grafted constellation.^39^ For the heterodimeric pair, inhibition depends on the presence of both partners in the coiled-coil assembly. For this new study, we added a suite of assemblies with different oligomerization states. Modification of the *a* and *d* positions in the heptad can be exploited to alter coiled-coil assembly state^21,32^ (Figure 1c,d). For instance substitution of *a* and *d* positions to either all isoleucine or alternating leucine and isoleucine residues were designed to produce parallel coiled-coil homotrimer CC-Tri_E1 and homotetramer CC-Tet_E1, respectively (Figure 1d). We selected the E1 rather than E2 graft for this series as a balance between the MCL-1 binding potency and coiled-coil stability. These peptides were anticipated to present three or four copies of the E1 motif on their outer surfaces, respectively. As a control, an analogous partly helical but monomeric peptide (Mono_E1) was generated by substituting the *a* and *d* positions to alanine,^48^ which should not form a stable coiled-coil assembly, but still presents the identified E1 binding motif.

### Modeling to the Probe the Peptide Assembly State and the Stoichiometries of Coiled-Coil Peptide/MCL-1 Complexes

AlphaFold2 (AF2) has emerged as a powerful tool to predict three-dimensional structures of proteins and protein/peptide complexes.^49–53^ AF2 was used to generate models of the individual coiled-coil scaffolds and their complexes with MCL-1 (Figure 2). For each scaffold and scaffold/protein complex, the average of relative predicted aligned error (pAE) and average per-residue model confidence score (pLDDT) were used to evaluate the confidence of assembly formation.^54,55^ Models were first generated with the peptide scaffold sequences alone, varying the total number of copies of the peptide from one to four as appropriate to assess the most stable oligomerization state of each sequence.

**Figure 2:**
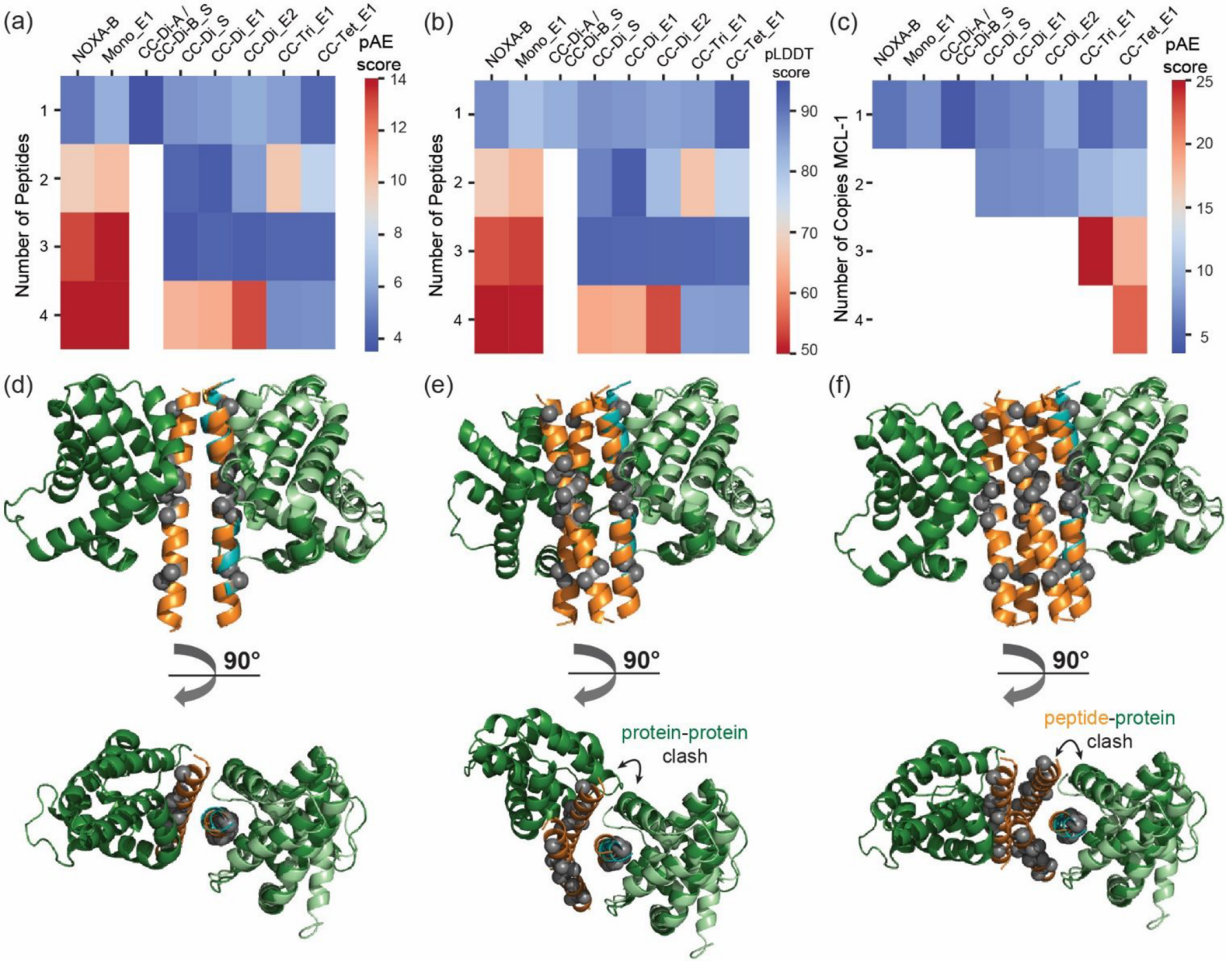
AlphaFold2 modeling of coiled coils and their complexes with MCL-1: (a) Each peptide sequence was modeled in AlphaFold2 (AF2), varying the total number of peptide copies with the average pAE score for the best model (lower values in blue indicate a better model); (b) Average pLDDT score for the best model (higher values in blue indicate a better model, data from the same prediction as (a)); (c) The complex of the expected oligomeric state and varying numbers of bound MCL-1 protein(s) was modeled with AF2 with the average pAE scores for the peptide residues shown (only the best model was used and lower values in blue indicate a better model); (d) AF2 model for the CC-Di_E1 dimer bound to two copies of MCL-1; (e) AF2 model for CC-Tri_E1 trimer bound to two copies of MCL-1 (steric clashes between MCL-1 protomers are evident); (f) AF2 model for CC-Tet_E1 tetramer bound to two copies of MCL-1 (steric clashes between MCL-1 and adjacent peptides in the tetramer are evident); coiled-coil peptides are shown in orange, hot-spot residues as gray spheres and MCL-1 in dark green; the model is overlaid with the NOXA-B:MCL-1 structure (PDB ID 2JM6, first model), where NOXA-B is shown in cyan, hot-spot residues in gray spheres and MCL-1 in light green.

The NOXA-B peptide modeled as a monomeric α helix and gave low pAE and high pLDDT scores, indicating this to be the preferred oligomerization state as expected (Figures 2a,b, S1 and S2). Mono_E1 was predicted to behave similarly. Modeling of the homodimeric coiled coils (CC-Di_S, CC-Di_E1, and CC-Di_E2) indicated the dimeric assemblies to be favorable, though trimers also scored favorably (Figure 2a,b). Although multiple oligomerization states can be observed for coiled-coil sequences,^56,57^ previous characterization of these homodimers by analytical ultracentrifugation (AUC) confirms dimeric assemblies.^39^ The heterodimeric coiled coil CC-Di-A/CC-Di-B_S gave low pAE and pLDDT scores for the 1:1 assembly. For both CC-Tri_E1 and CC-Tet_E1, monomeric, trimeric, and tetrameric assemblies were predicted to be accessible (Figure 2a,b). The targeted oligomer states all aligned well with crystal structures of the parent coiled-coil oligomers (Figure S3).

Next, models were generated for scaffolds in their designed oligomerization state (i.e., a coiled-coil dimer for dimeric sequences, a coiled-coil trimer for the trimeric sequence, etc.) bound to one through *n* copies of the MCL-1 protein (where *n* is the expected oligomerization state of the peptide) (examples in Figure 2d–f). These models predicted the most stable assemblies to be 1:1 peptide/protein complexes for NOXA-B and Mono_E1 (Figures 2c and S4a,b); 2:2 for homodimeric scaffolds (Figures 2c and S5c) and 1:1:1 for CC-Di-A/CC-Di-B_S (Figure S4c). Models of the complexes with CC-Tri_E1 suggest binding of 1 or potentially 2 copies of MCL-1 may be possible, but not 3 (Figures 2e and S6a) due to occlusion of the presented E1 binding motif and increased steric crowding as further copies of MCL-1 bind (see Tables S1 and S2). For CC-Tet_E1 (Figures 2c and S6b), the 4:1 peptide/protein assembly is most favorable, whereas the 4:2 (Figure 2f) and 4:3 assemblies are much less likely to form, and the 4:4 peptide/protein complex is unlikely to occur, again on steric grounds.

### Coiled-Coil Scaffolds Selectively Inhibit the BH3/MCL-1 Interaction

To assess the designs and models experimentally and to test their ability to act as oligomeric scaffolds for MCL-1 binding, peptides were prepared by Fmoc-based solid-phase peptide synthesis and purified by reversed-phase HPLC (see the Supporting Information for details and characterization).

Fluorescence anisotropy (FA) competition assays for BID/MCL-1 and BID/BCL-x_L_ were used to evaluate the inhibitory potencies.^58^ BCL-x_L_ is a further member of the BCL-2 family; BID recognizes both, whereas NOXA-B is selective for MCL-1.^59,60^ For BID/MCL-1, NOXA-B gave a potent inhibitory response (IC_50_ = 0.4 ± 0.1 μM), whereas no inhibition was observed with BID/BCL-x_L_ (Figure 3a) in line with the literature.^39,59,60^

**Figure 3:**
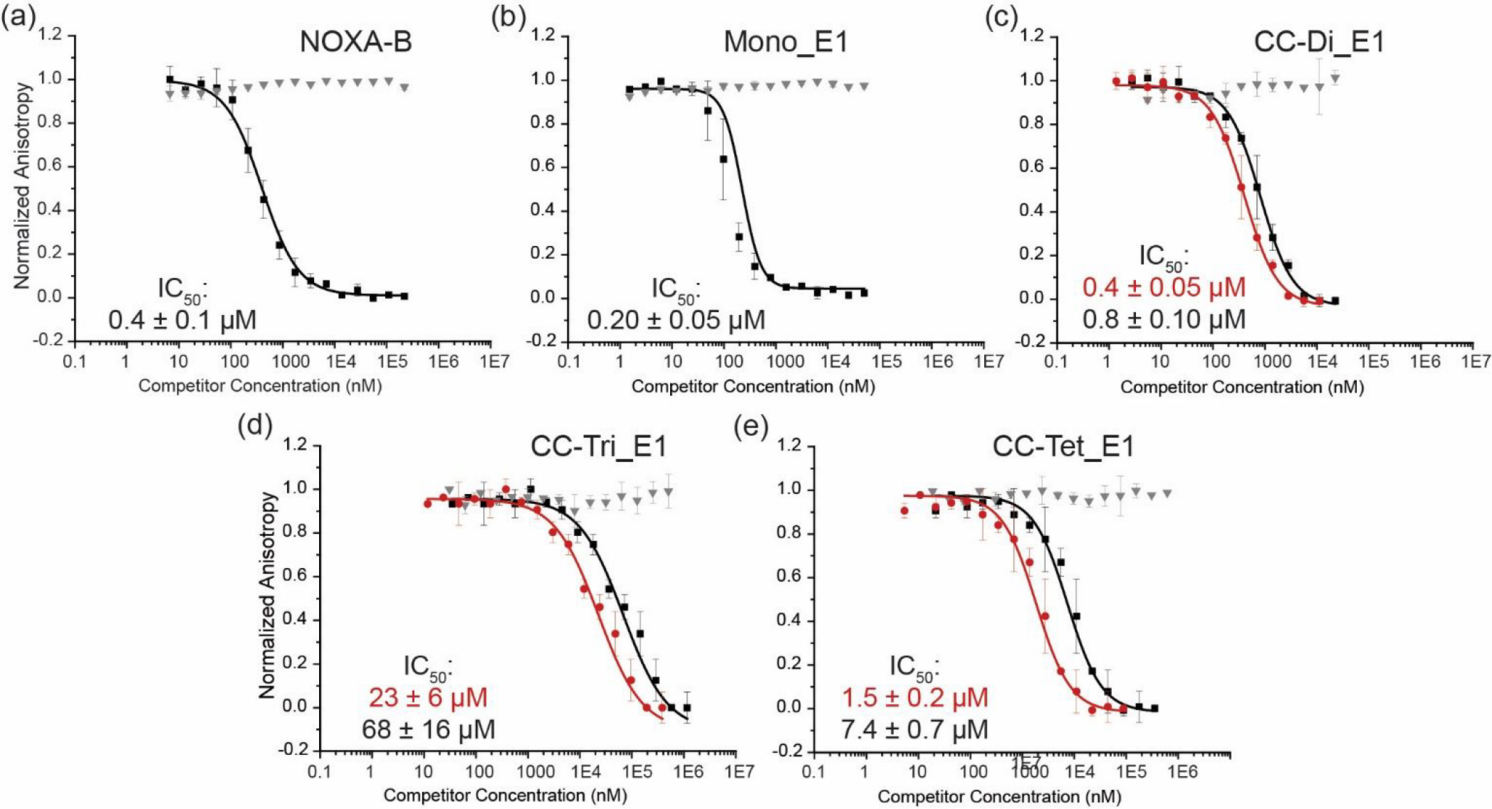
Fluorescence anisotropy competition assays of peptides: Titrations show the peptide constructs disrupting the FAM-Ahx-BID/MCL-1 complex (black squares, red circles), and testing against the FAM-Ahx-BID/BCL-x_L_ complex (gray triangles). Black squares show peptides disrupting FAM-Ahx-BID/MCL-1 with the competitor concentration normalized to the total peptide concentration (i.e., total number of grafted binding sites, to show binding relative to peptide concentration); red squares show the same data with the concentration normalized per scaffold’s designed oligomerization state; (a) NOXA-B; (b) CC-Mono_E1; (c) CC-Di_E1; (d) CC-Tri_E1; and (e) CC-Tet_E1 (150 nM MCL-1, 25 nM FAM-Ahx-BID, 20 °C, 50 mM Tris, 150 mM NaCl, pH 7.4, error bars from *n* = 3).

Consistent with our previous report,^39^ the undecorated parent homodimeric CC-Di peptide did not show a response in the BID/MCL-1 FA competition assay. Increasing the number of grafted hot-spot residues led to increased inhibition (CC-Di_S < CC-Di_E1 < CC-Di_E2, Figure S7a–c). Similarly,^39^ the 1:1 mixture of CC-Di-A and CC-Di-B_S showed inhibition (IC_50_ = 46 ± 5 μM, Figure S7d). None of these coiled-coil assemblies acted as BID/BCL-x_L_ inhibitors (Figure S7).

The monomeric control peptide, Mono_E1, was comparable in potency to those of NOXA-B and CC-Di_E1 (IC_50_ = 0.20 ± 0.05 μM, Figure 3b). This result may seem surprising. However, MCL-1 is known to recognize its ligands through a bind-and-fold mechanism.^58,61^ The high alanine content of Mono_E1 likely favors the MCL-1 bound helical conformation, and it contains eight hot-spot residues. Trimeric CC-Tri_E1 (IC_50_ = 68 ± 16 μM) and tetrameric CC-Tet_E1 IC_50_ = 7.4 ± 0.8 μM) were less potent BID/MCL-1 inhibitors (Figure 3d,e) than the dimer CC-Di_E1 (IC_50_ = 0.8 ± 0.1 μM). This behavior may reflect stepwise decreases in the accessible surface areas of the binding site with an increasing oligomerization state (Tables S1 and S2). In other words, higher-order coiled-coil assemblies occlude the grafted binding motifs, and, thus, coiled-coil oligomerization begins to compete with MCL-1 binding. This effect is likely compounded as increasing numbers of target proteins are recruited to the scaffolds; i.e., bound proteins will increasingly crowd the scaffold surface and further occlude the binding sites.

### Coiled coil/MCL-1 Complexes Display Hallmarks of Positively Cooperative, Mutually Stabilizing Interaction

CD spectroscopy was used to investigate the secondary structures and thermal stabilities of the designed peptides alone and in the presence of MCL-1. Both MCL-1 and all the coiled-coil assemblies gave CD spectra indicative of high helicity, while NOXA-B and Mono-E1 gave spectra consistent with disordered random coils (Figures S8a, S9 and S10a).^62^ Thermal denaturation of MCL-1 occurred with a midpoint *T*_m_ of 62 °C (Figure S7b and Table 1), comparable to published data.^63,64^

**Table 1:** Thermal Unfolding Properties for Coiled Coils in the Absence and Presence of MCL-1^a^

	*T*_m_ (°C)	*T*_m(pre)_ with MCL-1 (°C)	*T*_m_ with MCL-1 (°C)
MCL-1	62	N/A	N/A
NOXA-B	N/A	61	71
Mono_E1	N/A	61	73
CC-Di-A:CC-Di-B_S	78	67	72
CC-Di_S	53	60	72
CC-Di_E1	48	58	79
CC-Di_E2	31	34/65	82
CC-Tri_E1	∼60	65	72
CC-Tet_E1	n.d.	n.d.	78

a Conditions as for Figure 4.

The *T*_m_ for CC-Di_S, CC-Di_E1, and CC-Di_E2 decreased as the length of the grafted binding sequence increased (Table 1, Figure 4a–d).^39^ The monomeric peptides NOXA-B and Mono_E1, as expected, showed no unfolding transition (Figures 4e, S8b and Table 1). The *T*_m_ for CC-Tri_E1 was ∼60 °C (broad), and, for CC-Tet_E1 > 90 °C, which is comparable to the canonical *de novo* base CC-Tet coiled coil (Figure 4f,g and Table 1).^32^

**Figure 4:**
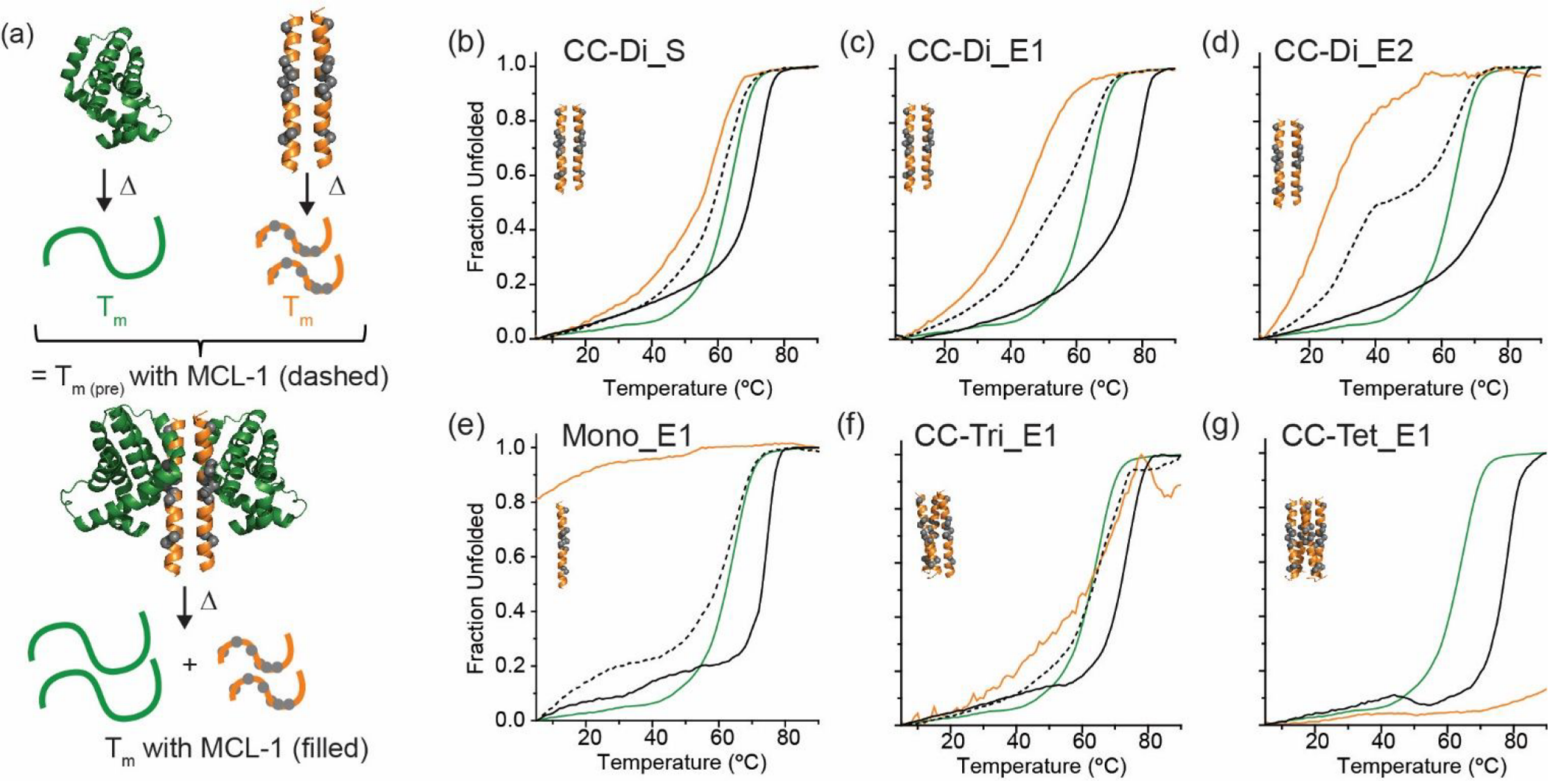
CD thermal unfolding curves for coiled-coil peptides, the protein MCL-1 and peptide/protein complexes: (a) schematic illustrating experimental workflow (for dimeric system), thermal unfolding of components (MCL-1 in green, monomeric/homomeric peptide samples in orange) are averaged to generate a theoretical unfolding curve for peptide/protein complexes (black dashes), and this is compared to the unfolding curve obtained for peptide/MCL-1 complex (black); (b) CC-Di_S (c) CC-Di_E1; (d) CC-Di_E2; (e) CC-Mono_E1 (f) CC-Tri_E1; and (g) CC-Tet_E1 (spectra monitored at λ = 222 nm, concentration = 25 μM of each component in 20 mM phosphate, 100 mM NaCl, pH 7.4).

Next, we performed CD analysis on the peptide/MCL-1 complexes. The stoichiometry was 1:1 grafted peptide:protein (e.g., for CC-Di_E1, 2:2 peptide/MCL-1 stoichiometry, for CC-Tri_E1, 3:3 peptide/MCL-1 stoichiometry for CC-Di-A/CC-Di-B_S/MCL-1 a 1:1:1 stoichiometry etc.). All peptide/MCL-1 samples were highly α-helical as expected (Figures S8a, S9 and S10a). Unfolding curves showed single transitions (Figures 4b–d and S10b) and gave *T*_m_ values shown in Table 1.

Thermal unfolding curves from the individual components were averaged to generate theoretical unfolding curves for the complex and predicted *T*_m(pre)_ values that assume no interaction between the components.^65^ The predicted values were compared with experimental values to determine whether complex formation is stabilizing. For all samples except CC-Tet_E1 (which does not unfold, precluding calculation of *T*_m(pre)_), the *T*_m_ values in the presence of MCL-1 were higher than predicted (Figure 4a–g, Table 1, Figures S8b and S10b). Most of the peptide–protein mixtures analyzed gave unfolding temperatures ∼10 °C higher than the calculated values. For CC-Di_E1 and CC-Di_E2, even higher values (∼20 °C) are observed in comparison to the predicted values. Collectively, these data provide confirmation of direct coiled coil/MCL-1 interaction and reveal that complex formation is mutually stabilizing for the monomeric, dimeric and trimeric coiled coils; i.e., binding stabilizes both the coiled-coil assembly and MCL-1 against disassembly and thermal denaturation^38^ (note: MCL-1 inhibitors have also been shown to suppress its unfolding).^63^ The similarity in *T*_m_ for the monomeric, trimeric, and tetrameric samples in the presence of MCL-1 may indicate that interaction between CC-Tri_E1 or Tet_E1 and MCL-1 occurs at 1:1 stoichiometry (i.e., the oligomer dissociates in favor of MCL-1 binding, see later).

### Coiled Coils Can Recruit Multiple Copies of the Target Protein

Complexes of the coiled-coil scaffolds with MCL-1 were evaluated by native mass spectrometry (Native MS)^66^ and analytical centrifugation (AUC) to assess oligomerization in the bound states.

Native MS of coiled coils is challenging given that their assembly is driven through hydrophobicity, which is weakened when samples are transferred to the gas-phase in MS,^67^ although assembled coiled coils can be observed under certain ionization conditions.^68^ Native MS analysis for CC-Di_S/MCL-1 indicated a mixture of unbound peptide, unbound protein, and peptide/protein complexes, with relatively larger amounts of free peptide and protein than complex (Figure 5b). For CC-Di_S/MCL-1, both the 1:1 and 2:2 complexes were observed, with slightly more 2:2 complex present (Figure 5b). For CC-Di_E1 and CC-Di_E2, the 2:2 peptide/protein complexes were the dominant species (Figure 5c,d). These data correlate with more potent inhibitory activities. Analysis of heterodimer CC-Di-A:CC-Di-B_S with MCL-1 revealed a relatively large amount of unbound peptide and protein (Figure 5e), together with coiled coil/MCL-1 complexes of varied stoichiometry and composition, which, is consistent with the weaker binding potency. CC-Tri_E1 could not be analyzed because of the tendency of the peptide to precipitate. For CC-Tet_E1/MCL-1 large amounts of free MCL-1 were detected alongside free monomeric peptide. 1:1 and 2:2 complexes were the only peptide/protein complexes detected and no tetrameric peptide scaffold alone or associated with 1 to 4 copies of MCL-1 were observed (Figure 5f). The fact that the 2:2 complex was observed and the coiled coil alone is absent from the mass spectra suggests that MCL-1:MCL-1 interactions aid in complex stabilization.

**Figure 5:**
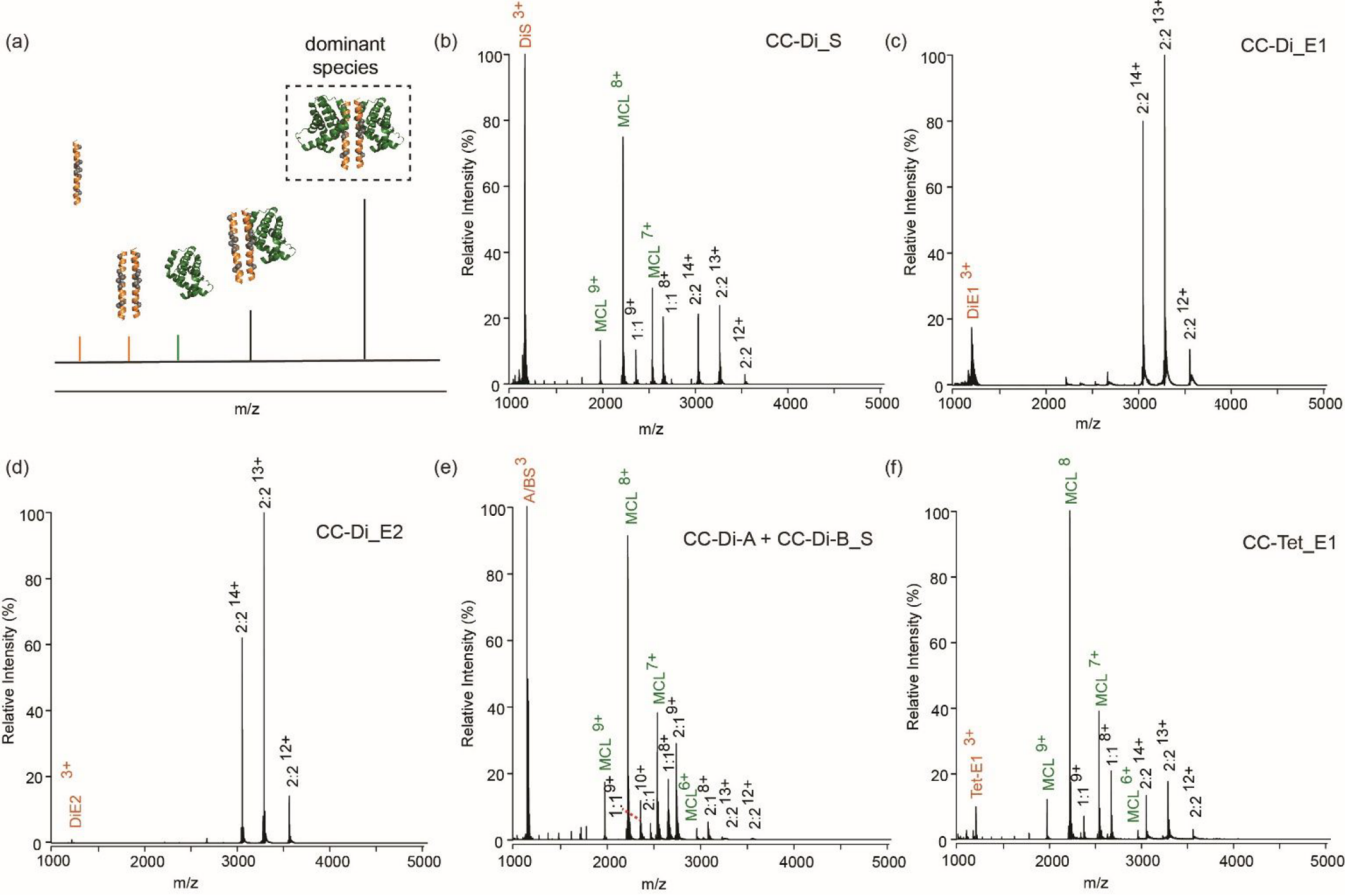
Native mass spectrometry analysis of coiled-coil peptides with MCL-1 protein: (a) schematic illustrating how ESI MS data are used to assess the dominant speciation in stoichiometric mixtures of peptide and MCL-1 (orange indicates peaks corresponding to peptide alone, green indicates MCL-1 protein peaks and black indicates peaks corresponding to complexes, with the stoichiometries displayed in panels (b–f); (b) CC-Di_S; (c) CC-Di_E1; (d) CC-Di_E2; (e) CC-Di-A:CC-Di-B_S (1:1 label represents CC-Di-A/MCL-1 and CC-Di-B_S/MCL-1 [peaks overlapping], 2:1 label represents CC-Di-A/CC-Di-B_S/MCL-1, 2:2 label represents 2 × CC-Di-A/2 X MCL-1 and 2 × CC-Di-B_S/2 × MCL-1 and 2 × CC-Di-A/CC-Di-B_S/MCL-1); and (f) CC-Tet_E1 (samples analyzed at 5 μM of each component).

To explore the oligomerization states further, we performed sedimentation velocity (SV) analytical ultracentrifugation (AUC) on the MCL-1 with peptide mixtures (Table S3 and Figure S11). For an equimolar mixture of CC-Di_E1 and MCL-1, the fitting of the sedimentation data indicated that the 2:2 complex was the only state present. At higher peptide concentration, the sedimentation peak broadened and shifted to a smaller sedimentation coefficient, consistent with the presence of smaller oligomers; however, the fitted molecular weight was larger than a 2:1 CC-Di_E1:MCL-1 stoichiometry, indicating the 2:2 complex remained. This is consistent with positive cooperative formation of a ternary complex, as indicated by the CD and native MS data. For CC-Tri_E1, a small shift in the peak compared to MCL-1 was observed with the fitted molecular weight, indicating peptide binding. At higher peptide concentration, a broadened peak indicated multiple species. For CC-Tet_E1, a peak with fitted mass between 4:1 CC-Tet_E1:MCL-1 and 2:2 CC-Tet_E1:MCL-1 was observed. In addition, for the equimolar mixture of CC-Tet_E1 and MCL-1, a small peak corresponding to the weight of MCL-1 was observed alongside an even smaller peak for a higher weight. At increased peptide ratios, a peak corresponding to a small weight appeared, likely an unbound CC-Tet_E1 tetramer. Further size exclusion chromatography (SEC) experiments for the MCL-1 plus peptide mixtures (Figure S12) confirmed these observations.

Overall, native MS data and AUC and SEC data are broadly consistent across the coiled-coil series. Data for the homodimers show they cooperatively recruit and bind two copies of MCL-1. AF2 modeling suggests this is possible, and this is compatible with our interpretation of the unfolding experiments (*T*_m(dimer/MCL‑1)_ > *T*_m(monomer/MCL1)_). For trimeric and tetrameric coiled coils, the data are more ambiguous. AF2 modeling suggests that binding of multiple copies of MCL-1 to trimer and tetramer is sterically constrained. Higher-order protein-peptide oligomers were not abundant in native MS, AUC, or SEC experiments. Moreover, the inhibitory potencies for both are lower than monomeric controls, while unfolding of complexes is comparable (*T*_m(oligomer/MCL‑1)_ ∼ *T*_m(monomer/MCL‑1)_). This is consistent with a more complex equilibrium where larger oligomers are able to recruit fewer copies of MCL-1, and so to maximize interactions, peptide/MCL-1 binding competes with coiled-coil oligomerization.

## Conclusions

Here, we have explored the grafting of hot-spot binding residues onto coiled-coil peptide assemblies and investigated the binding behavior of these peptides toward MCL-1. We grafted binding sequences onto monomeric, homo- and heterodimeric, homotrimeric, and homotetrameric sequences in order to test the potential of multivalency to enhance the affinity. Our data suggest that grafted dimeric peptide scaffolds have the optimal balance of potency, stability, fidelity to the intended peptide assembly state, and the ability to recruit multiple (two) copies of protein. Indeed, they do not dissociate to form 1:1 peptide/MCL-1 complexes; rather, they exhibit a strong propensity to form high-affinity 2:2 peptide/MCL-1 ternary complexes with positive cooperativity. This results in mutual stabilization of the protein-target and the coiled coil, consistent with positive cooperative binding. For higher-order trimeric and tetrameric coiled-coil designs, the reduced inhibitory potency likely arises from a reduction in the accessibility of the binding motif in these scaffolds relative to the monomeric and dimeric scaffolds.

In summary, we have developed grafted multivalent coiled-coil peptides for the selective inhibition of the antiapoptotic cancer protein MCL-1, which can potentially serve as therapeutic PPI inhibitors. For the future, our study indicates that grafting and multivalent coiled-coil strategies might be most effective using dimeric scaffolds. Nonetheless, this offers the prospect of designing homo- and heterodimeric coiled coils that recruit two copies of one protein target or two different proteins, for example, a target and effector, to act as “molecular glues” for proximity-induced pharmacology. For molecular glues–cooperative ternary complex formation is considered desirable for proteolytic degradation, and our data indicate that the effect of ternary complex formation on folding stability also warrants consideration.

## Supplementary Material



## Data Availability

Supporting mass spectrometry raw data files are openly available from the University of Birmingham data archive at 10.25500/edata.bham.00001286.

## Notes

Additional data figures, experimental methods and details, peptide and protein characterization data (PDF)

## Abbreviations

PPI: protein–protein interaction
BCL-2: B-cell lymphoma 2
MOMP: mitochondrial outer membrane permeabilization
pAE: predicted aligned error
pLDDT: per-residue model confidence score
FA: fluorescence anisotropy
IC_50_: half-maximal inhibitory concentration
CD: circular dichroism
AUC: analytical ultracentrifugation
MS: mass spectrometry
AF2: AlphaFold2

## References

[1] Nooren I. M. A., Thornton J. M. (2003). Diversity of Protein-Protein Interactions. EMBO J..

[2] Braun P., Gingras A. C. (2012). History of protein-protein
interactions: from egg-white
to complex networks. Proteomics.

[3] Skinnider M. A., Scott N. E., Prudova A., Kerr C. H., Stoynov N., Stacey R. G., Chan Q. W. T., Rattray D., Gsponer J., Foster L. J. (2021). An atlas of protein-protein
interactions across mouse
tissues. Cell.

[4] Arkin M. R., Tang Y., Wells J. A. (2014). Small-molecule inhibitors
of protein-protein
interactions: progressing toward the reality. Chem. Biol..

[5] Lu H., Zhou Q., He J., Jiang Z., Peng C., Tong R., Shi J. (2020). Recent advances
in the development
of protein-protein interactions modulators: mechanisms and clinical
trials. Signal Transduct. Target. Ther..

[6] Wang H., Dawber R. S., Zhang P., Walko M., Wilson A. J., Wang X. (2021). Peptide-based inhibitors of protein-protein
interactions: biophysical,
structural and cellular consequences of introducing a constraint. Chem. Sci..

[7] Palomo J. M. (2014). Solid-phase
peptide synthesis: an overview focused on the preparation of biologically
relevant peptides. RSC Adv..

[8] Listov D., Goverde C. A., Correia B. E., Fleishman S. J. (2024). Opportunities
and challenges in design and optimization of protein function. Nat. Rev. Mol. Cell Biol..

[9] Chu A. E., Lu T., Huang P.-S. (2024). Sparks
of function by de novo protein design. Nat.
Biotechnol..

[10] Kortemme T. (2024). De novo protein
design—From new structures to programmable functions. Cell.

[11] Berger S., Procko E., Margineantu D., Lee E. F., Shen B. W., Zelter A., Silva D. A., Chawla K., Herold M. J., Garnier J. M., Johnson R., MacCoss M. J., Lessene G., Davis T. N., Stayton P. S., Stoddard B. L., Fairlie W. D., Hockenbery D. M., Baker D. (2016). Computationally designed high specificity
inhibitors delineate the roles of BCL2 family proteins in cancer. eLife.

[12] Chevalier A., Silva D.-A., Rocklin G. J., Hicks D. R., Vergara R., Murapa P., Bernard S. M., Zhang L., Lam K.-H., Yao G., Bahl C. D., Miyashita S.-I., Goreshnik I., Fuller J. T., Koday M. T., Jenkins C. M., Colvin T., Carter L., Bohn A., Bryan C. M., Fernández-Velasco D. A., Stewart L., Dong M., Huang X., Jin R., Wilson I. A., Fuller D. H., Baker D. (2017). Massively parallel
de novo protein design for targeted therapeutics. Nature.

[13] Adihou H., Gopalakrishnan R., Förster T., Guéret S. M., Gasper R., Geschwindner S., Carrillo García C., Karatas H., Pobbati A. V., Vazquez-Chantada M., Davey P., Wassvik C. M., Pang J. K. S., Soh B. S., Hong W., Chiarparin E., Schade D., Plowright A. T., Valeur E., Lemurell M., Grossmann T. N., Waldmann H. (2020). A protein tertiary structure mimetic modulator of the
Hippo signalling pathway. Nat. Commun..

[14] Pannecoucke E., Van Trimpont M., Desmet J., Pieters T., Reunes L., Demoen L., Vuylsteke M., Loverix S., Vandenbroucke K., Alard P., Henderikx P., Deroo S., Baatz F., Lorent E., Thiolloy S., Somers K., McGrath Y., Vlierberghe P. V., Lasters I., Savvides S. N. (2021). Cell-penetrating
Alphabody protein scaffolds for intracellular drug targeting. Sci. Adv..

[15] Cao L., Coventry B., Goreshnik I., Huang B., Sheffler W., Park J. S., Jude K. M., Marković I., Kadam R. U., Verschueren K. H. G., Verstraete K., Walsh S. T. R., Bennett N., Phal A., Yang A., Kozodoy L., DeWitt M., Picton L., Miller L., Strauch E.-M., DeBouver N. D., Pires A., Bera A. K., Halabiya S., Hammerson B., Yang W., Bernard S., Stewart L., Wilson I. A., Ruohola-Baker H., Schlessinger J., Lee S., Savvides S. N., Garcia K. C., Baker D. (2022). Design of protein-binding proteins
from the target structure alone. Nature.

[16] Torres S. V., Leung P. J. Y., Venkatesh P., Lutz I. D., Hink F., Huynh H.-H., Becker J., Yeh A. H.-W., Juergens D., Bennett N. R., Hoofnagle A. N., Huang E., MacCoss M. J., Expòsit M., Lee G. R., Bera A. K., Kang A., De La Cruz J., Levine P. M., Li X., Lamb M., Gerben S. R., Murray A., Heine P., Korkmaz E. N., Nivala J., Stewart L., Watson J. L., Rogers J. M., Baker D. (2024). De novo design
of high-affinity binders of bioactive helical peptides. Nature.

[17] Swanson S., Sivaraman V., Grigoryan G., Keating A. E. (2022). Tertiary motifs
as building blocks for the design of protein-binding peptides. Protein Sci..

[18] Dauparas J., Anishchenko I., Bennett N., Bai H., Ragotte R. J., Milles L. F., Wicky B. I. M., Courbet A., de Haas R. J., Bethel N., Leung P. J. Y., Huddy T. F., Pellock S., Tischer D., Chan F., Koepnick B., Nguyen H., Kang A., Sankaran B., Bera A. K., King N. P., Baker D. (2022). Robust deep
learning–based protein sequence design using ProteinMPNN. Science.

[19] Watson J. L., Juergens D., Bennett N. R., Trippe B. L., Yim J., Eisenach H. E., Ahern W., Borst A. J., Ragotte R. J., Milles L. F., Wicky B. I. M., Hanikel N., Pellock S. J., Courbet A., Sheffler W., Wang J., Venkatesh P., Sappington I., Torres S. V., Lauko A., De Bortoli V., Mathieu E., Ovchinnikov S., Barzilay R., Jaakkola T. S., DiMaio F., Baek M., Baker D. (2023). De novo design of protein
structure and function with RFdiffusion. Nature.

[20] Pacesa, M. ; Nickel, L. ; Schellhaas, C. ; Schmidt, J. ; Pyatova, E. ; Kissling, L. ; Barendse, P. ; Choudhury, J. ; Kapoor, S. ; Alcaraz-Serna, A. ; Cho, Y. ; Ghamary, K. H. ; Vinué, L. ; Yachnin, B. J. ; Wollacott, A. M. ; Buckley, S. ; Westphal, A. H. ; Lindhoud, S. ; Georgeon, S. ; Goverde, C. A. ; Hatzopoulos, G. N. ; Gönczy, P. ; Muller, Y. D. ; Schwank, G. ; Swarts, D. C. ; Vecchio, A. J. ; Schneider, B. L. ; Ovchinnikov, S. ; Correia, B. E. , BindCraft: one-shot design of functional protein binders. bioRxiv 2024 10.1101/2024.09.30.615802. PMC12507698 40866699

[21] Woolfson D. N. (2023). Understanding
a protein fold: The physics, chemistry, and biology of alpha-helical
coiled coils. J. Biol. Chem..

[22] Thompson K. E., Bashor C. J., Lim W. A., Keating A. E. (2012). SYNZIP Protein Interaction
Toolbox: in Vitro and in Vivo Specifications of Heterospecific Coiled-Coil
Interaction Domains. ACS Synth. Biol..

[23] Lee J.-H., Kang E., Lee J., Kim J., Lee K. H., Han J., Kang H. Y., Ahn S., Oh Y., Shin D., Hur K., Chae S. Y., Song P. H., Kim Y.-I., Park J. C., Lee J. I. (2014). Protein grafting
of p53TAD onto a leucine zipper scaffold
generates a potent HDM dual inhibitor. Nat.
Commun..

[24] Sadek J., Wuo M. G., Rooklin D., Hauenstein A., Hong S. H., Gautam A., Wu H., Zhang Y., Cesarman E., Arora P. S. (2020). Modulation of virus-induced
NF-κB
signaling by NEMO coiled coil mimics. Nat. Commun..

[25] Rhys G. G., Cross J. A., Dawson W. M., Thompson H. F., Shanmugaratnam S., Savery N. J., Dodding M. P., Hocker B., Woolfson D. N. (2022). De novo
designed peptides for cellular delivery and subcellular localisation. Nat. Chem. Biol..

[26] Smith A. J., Naudin E. A., Edgell C. L., Baker E. G., Mylemans B., FitzPatrick L., Herman A., Rice H. M., Andrews D. M., Tigue N., Woolfson D. N., Savery N. J. (2023). Design
and Selection
of Heterodimerizing Helical Hairpins for Synthetic Biology. ACS Synth. Biol..

[27] Makri
Pistikou A.-M., Cremers G. A. O., Nathalia B. L., Meuleman T. J., Bögels B. W. A., Eijkens B. V., de Dreu A., Bezembinder M. T. H., Stassen O. M. J. A., Bouten C. C. V., Merkx M., Jerala R., de Greef T. F. A. (2023). Engineering a scalable and orthogonal
platform for synthetic communication in mammalian cells. Nat. Commun..

[28] Cross J. A., Dawson W. M., Shukla S. R., Weijman J. F., Mantell J., Dodding M. P., Woolfson D. N. (2024). A de novo
designed coiled coil-based
switch regulates the microtubule motor kinesin-1. Nat. Chem. Biol..

[29] Britton D., Katsara O., Mishkit O., Wang A., Pandya N., Liu C., Mao H., Legocki J., Jia S., Xiao Y., Aristizabal O., Paul D., Deng Y., Schneider R., Wadghiri Y. Z., Montclare J. K. (2024). Engineered
coiled-coil HIF1α
protein domain mimic. Biomater. Sci..

[30] Utterstrom J., Naeimipour S., Selegard R., Aili D. (2021). Coiled coil-based therapeutics
and drug delivery systems. Adv. Drug. Delivery
Rev..

[31] Plaper T., Rihtar E., Železnik Ramuta T., Forstnerič V., Jazbec V., Ivanovski F., Benčina M., Jerala R. (2024). The art of designed coiled-coils for the regulation
of mammalian cells. Cell Chem. Biol..

[32] Fletcher J. M., Boyle A. L., Bruning M., Bartlett G. J., Vincent T. L., Zaccai N. R., Armstrong C. T., Bromley E. H., Booth P. J., Brady R. L., Thomson A. R., Woolfson D. N. (2012). A basis set of de
novo coiled-coil peptide oligomers for rational protein design and
synthetic biology. ACS Synth. Biol..

[33] Dawson W. M., Martin F. J. O., Rhys G. G., Shelley K. L., Brady R. L., Woolfson D. N. (2021). Coiled coils 9-to-5:
rational de novo design of alpha-helical
barrels with tunable oligomeric states. Chem.
Sci..

[34] Hilditch A.
T., Romanyuk A., Cross S. J., Obexer R., McManus J. J., Woolfson D. N. (2024). Assembling
membraneless organelles from de novo designed
proteins. Nat. Chem..

[35] Mammen M., Choi S.-K., Whitesides G. M. (1998). Polyvalent
Interactions in Biological
Systems: Implications for Design and Use of Multivalent Ligands and
Inhibitors. Angew. Chem., Int. Ed..

[36] Kiessling L. L., Gestwicki J. E., Strong L. E. (2000). Synthetic Multivalent Ligands in
the Exploration of Cell-Surface Interactions. Curr. Opin. Chem. Biol..

[37] Diamante A., Chaturbedy P. K., Rowling P. J. E., Kumita J. R., Eapen R. S., McLaughlin S. H., de la Roche M., Perez-Riba A., Itzhaki L. S. (2021). Engineering mono-
and multi-valent inhibitors on a
modular scaffold. Chem. Sci..

[38] Williams D. H., Stephens E., O’Brien D. P., Zhou M. (2004). Understanding Noncovalent
Interactions: Ligand Binding Energy and Catalytic Efficiency from
Ligand-Induced Reductions in Motion within Receptors and Enzymes. Angew. Chem., Int. Ed..

[39] Fletcher J. M., Horner K. A., Bartlett G. J., Rhys G. G., Wilson A. J., Woolfson D. N. (2018). De novo coiled-coil
peptides as scaffolds for disrupting
protein-protein interactions. Chem. Sci..

[40] Cory S., Adams J. M. (2002). The BCL2 family:
Regulators of the cellular life-or-death
switch. Nat. Rev. Canc..

[41] Czabotar P. E., Garcia-Saez A. J. (2023). Mechanisms
of BCL-2 family proteins in mitochondrial
apoptosis. Nat. Rev. Mol. Cell Biol..

[42] Singh R., Letai A., Sarosiek K. (2019). Regulation
of apoptosis in health
and disease: the balancing act of BCL-2 family proteins. Nat. Rev. Mol. Cell Biol..

[43] Osterlund E. J., Hirmiz N., Pemberton J. M., Nougarède A., Liu Q., Leber B., Fang Q., Andrews D. W. (2022). Efficacy and specificity
of inhibitors of BCL-2 family protein interactions assessed by affinity
measurements in live cells. Sci. Adv..

[44] Hird A. W., Tron A. E. (2019). Recent advances in the development
of Mcl-1 inhibitors
for cancer therapy. Pharmacol. Ther..

[45] Konstantinidou M., Arkin M. R. (2024). Molecular glues
for protein-protein interactions: Progressing
toward a new dream. Cell Chem. Biol..

[46] Barile E., Marconi G. D., De S. K., Baggio C., Gambini L., Salem A. F., Kashyap M. K., Castro J. E., Kipps T. J., Pellecchia M. (2017). hBfl-1/hNOXA
Interaction Studies Provide New Insights
on the Role of Bfl-1 in Cancer Cell Resistance and for the Design
of Novel Anticancer Agents. ACS Chem. Biol..

[47] Woolfson D. N. (2005). The Design
of Coiled-Coil Structures and Assemblies. Adv.
Protein Chem..

[48] Leonard D. J., Zieleniewski F., Wellhöfer I., Baker E. G., Ward J. W., Woolfson D. N., Clayden J. (2021). Scalable synthesis and coupling of
quaternary α-arylated amino acids: α-aryl substituents
are tolerated in α-helical peptides. Chem.
Sci..

[49] Jumper J., Evans R., Pritzel A., Green T., Figurnov M., Ronneberger O., Tunyasuvunakool K., Bates R., Zidek A., Potapenko A., Bridgland A., Meyer C., Kohl S. A. A., Ballard A. J., Cowie A., Romera-Paredes B., Nikolov S., Jain R., Adler J., Back T., Petersen S., Reiman D., Clancy E., Zielinski M., Steinegger M., Pacholska M., Berghammer T., Bodenstein S., Silver D., Vinyals O., Senior A. W., Kavukcuoglu K., Kohli P., Hassabis D. (2021). Highly accurate protein
structure prediction with AlphaFold. Nature.

[50] Yin R., Feng B. Y., Varshney A., Pierce B. G. (2022). Benchmarking AlphaFold
for protein complex modeling reveals accuracy determinants. Protein Sci..

[51] Tsaban T., Varga J. K., Avraham O., Ben-Aharon Z., Khramushin A., Schueler-Furman O. (2022). Harnessing
protein folding neural
networks for peptide-protein docking. Nat. Commun..

[52] Mirdita M., Schutze K., Moriwaki Y., Heo L., Ovchinnikov S., Steinegger M. (2022). ColabFold: making protein folding
accessible to all. Nat. Methods.

[53] Bryant P. (2023). Deep learning
for protein complex structure prediction. Curr.
Opin. Struct. Biol..

[54] Mariani V., Biasini M., Barbato A., Schwede T. (2013). lDDT: a local
superposition-free
score for comparing protein structures and models using distance difference
tests. Bioinformatics.

[55] Varadi M., Anyango S., Deshpande M., Nair S., Natassia C., Yordanova G., Yuan D., Stroe O., Wood G., Laydon A., Zidek A., Green T., Tunyasuvunakool K., Petersen S., Jumper J., Clancy E., Green R., Vora A., Lutfi M., Figurnov M., Cowie A., Hobbs N., Kohli P., Kleywegt G., Birney E., Hassabis D., Velankar S. (2022). AlphaFold Protein Structure Database:
massively expanding the structural coverage of protein-sequence space
with high-accuracy models. Nucleic. Acids. Res..

[56] Oshaben K. M., Salari R., McCaslin D. R., Chong L. T., Horne W. S. (2012). The native
GCN4 leucine-zipper domain does not uniquely specify a dimeric oligomerization
state. Biochemistry.

[57] Fletcher J. M., Bartlett G. J., Boyle A. L., Danon J. J., Rush L. E., Lupas A. N., Woolfson D. N. (2017). N@a and
N@d: Oligomer and Partner
Specification by Asparagine in Coiled-Coil Interfaces. ACS Chem. Biol..

[58] Miles J. A., Yeo D. J., Rowell P., Rodriguez-Marin S., Pask C. M., Warriner S. L., Edwards T. A., Wilson A. J. (2016). Hydrocarbon
constrained peptides - understanding preorganisation and binding affinity. Chem. Sci..

[59] Chen L., Willis S. N., Wei A., Smith B. J., Fletcher J. I., Hinds M. G., Colman P. M., Day C. L., Adams J. M., Huang D. C. (2005). Differential targeting of prosurvival Bcl-2 proteins
by their BH3-only ligands allows complementary apoptotic function. Mol. Cell.

[60] Certo M., Moore V. D. G., Nishino M., Wei G., Korsmeyer S., Armstrong S. A., Letai A. (2006). Mitochondria primed
by death signals
determine cellular addiction to antiapoptotic BCL-2 family members. Cancer Cell.

[61] Rogers J. M., Wong C. T., Clarke J. (2014). Coupled Folding
and Binding of the
Disordered Protein PUMA Does Not Require Particular Residual Structure. J. Am. Chem. Soc..

[62] Greenfield N. J. (2006). Using circular
dichroism spectra to estimate protein secondary structure. Nat. Protoc..

[63] Milani M., Byrne D. P., Greaves G., Butterworth M., Cohen G. M., Eyers P. A., Varadarajan S. (2018). DRP-1 is required
for BH3 mimetic-mediated mitochondrial fragmentation and apoptosis. Cell Death Dis.

[64] Craxton A., Butterworth M., Harper N., Fairall L., Schwabe J., Ciechanover A., Cohen G. M. (2012). NOXA, a sensor of proteasome integrity,
is degraded by 26S proteasomes by an ubiquitin-independent pathway
that is blocked by MCL-1. Cell Death Differ..

[65] Note: The distinct transitions in the predicted curve for the mixture with CC-Di_E2 reflects the two very different unfolding profiles and Tm values for the individual components.

[66] Leney A. C., Heck A. J. R. (2017). Native Mass Spectrometry: What is
in the Name?. J. Am. Soc. Mass Spectrom..

[67] Hernández H., Robinson C. V. (2007). Determining the stoichiometry and interactions of macromolecular
assemblies from mass spectrometry. Nat. Protoc..

[68] Cristie-David A. S., Sciore A., Badieyan S., Escheweiler J. D., Koldewey P., Bardwell J. C. A., Ruotolo B. T., Marsh E. N. G. (2017). Evaluation
of de novo-designed coiled coils as off-the-shelf components for protein
assembly. Mol. Sys. Des. Eng..

