# Supplementary material for: Grafted Coiled-Coil Peptides as Multivalent Scaffolds for Protein Recognition
Source: ACS Chem Biol. 2025 Jun 5;20(6):1309–18. doi: 10.1021/acschembio.5c00137 (PMC12186253; doi:10.1021/acschembio.5c00137)
Supplement: Supplementary file 1 [file cb5c00137_si_001.pdf]

**Supporting Information for:**

# Grafted Coiled-Coil Peptides as Multivalent Scaffolds for Protein Recognition


Amanda M. Acevedo-Jake[1], Bram Mylemans[2], Danielle F. Kay[3], Peiyu Zhang[4], Boguslawa Korona[5], Guto G. Rhys,[6] Aneika C. Leney[3], Danny T. Huang,[7,8] Thomas A. Edwards[9], Laura S. Itzhaki[5], Derek N. Woolfson[2,10,11], Andrew J. Wilson[1]

*Author Affiliations*

[1]School of Chemistry, University of Birmingham, Edgbaston, B15 2TT, UK

[2]School of Chemistry, University of Bristol, Cantock's Close, Bristol BS8 1TS, UK

[3]School of Biosciences, University of Birmingham, Edgbaston B15 2TT, UK

[4]School of Chemistry, University of Leeds, Woodhouse Lane, Leeds LS2 9JT, UK

[5]Department of Pharmacology, University of Cambridge, Cambridge CB2 1PD, UK

[6]School of Chemistry, Cardiff University, Main Building, Park Place, Cardiff, CF10 3AT

[7]Cancer Research UK Scotland Institute, Garscube Estate, Switchback Road, Glasgow G61 1BD, UK

[8]School of Cancer Sciences, University of Glasgow, Garscube Estate, Switchback Road, Glasgow G61 1QH, UK

[9]College of Biomedical Sciences, Larkin University, 18301 N Miami Ave #1, Miami, Florida, 33169 USA

[10]School of Biochemistry, University of Bristol, Medical Sciences Building, University Walk, Bristol BS8 1TD, UK

[11]BrisSynBio, University of Bristol, Life Sciences Building, Tyndall Avenue, Bristol, BS8 1TQ, UK

**Table of Contents**

Supplementary Figures ........ 2
AlphaFold2 Modelling ........ 14
Protein Overexpression and Purification ........ 14
Fluorescence Anisotropy Competition Assays ........ 18
Circular Dichroism ........ 19
Native Mass Spectrometry Analysis ........ 19
Peptide Synthesis ........ 21
Analytical HPLC and MS of peptides ........ 23
References ........ 32

**Supplementary Figures**

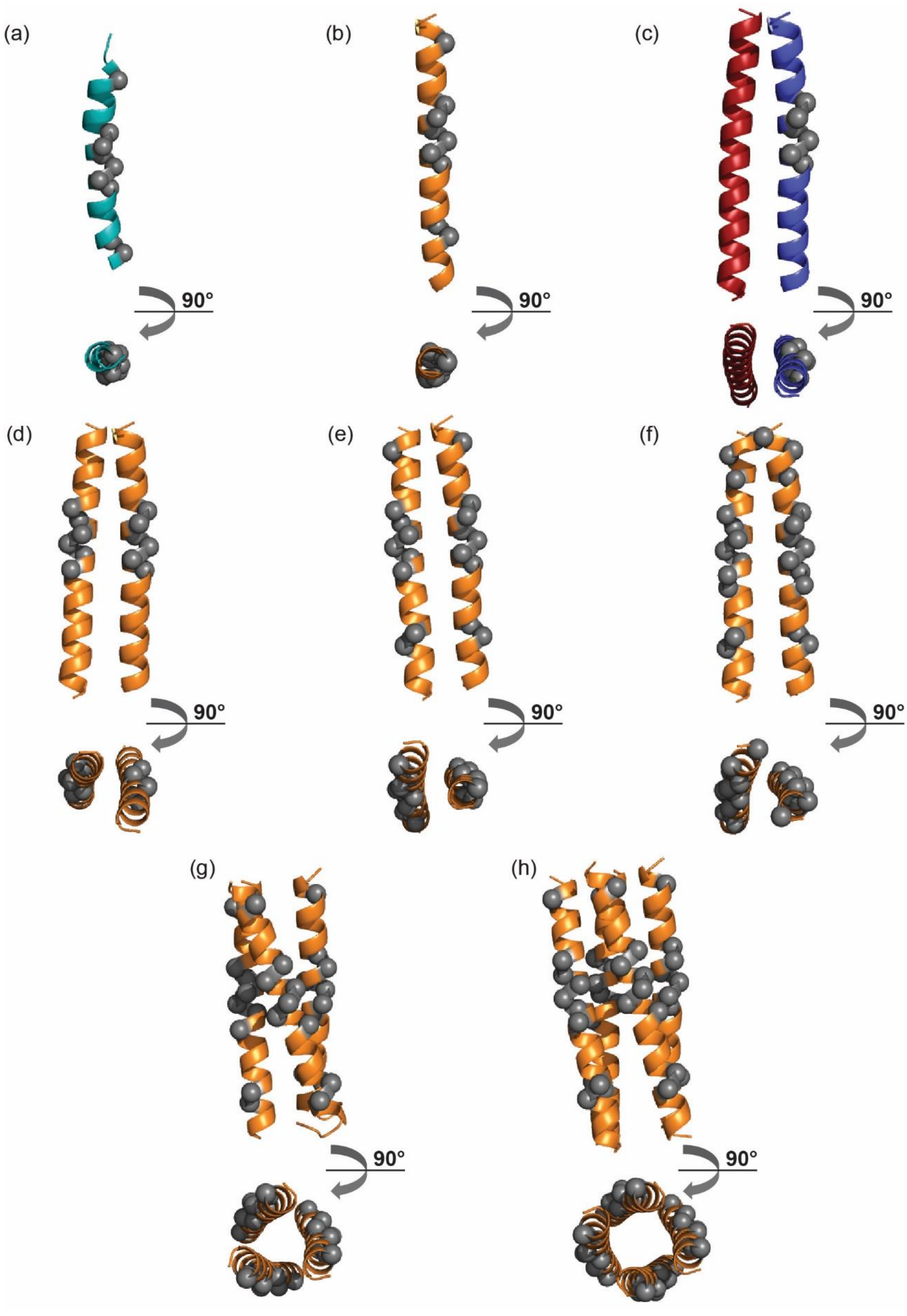


**Figure S1. NOXA-B and models of coiled-coil peptide scaffolds alone:** models generated from AF2, corresponding to the heat map data shown in Figure 2 and Figure S2. Panels are: (a) NOXA-B (from PDB ID 2JM6, model 1); (b) Mono_E1; (c) CC-Di-A:CC-Di-B_S; (d) CC-Di_S; (e) CC-Di_E1; (f) CC-Di_E2; (g) CC-Tri_E1; and, (h) CC-Tet_E1,

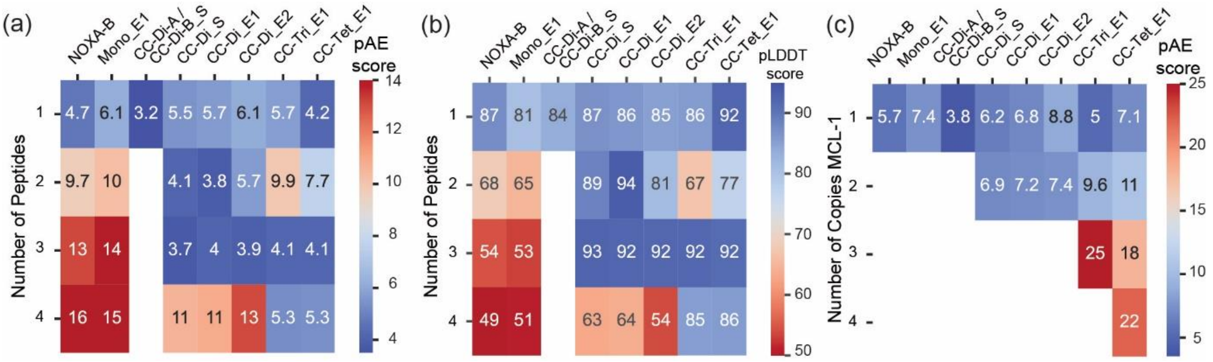


**Figure S2. AlphaFold2 modelling of coiled-coils and their complexes with MCL-1** (reproduced here from Figure 2 with numerical values included): (a) Each peptide sequence was modelled in AlphaFold2, varying the total number of peptide copies with the average pAE score for the best model shown (lower values in blue indicate a better model); (b) Average pLDDT score for the best model is shown (higher values in blue indicate a better model, data from the same prediction as (a)); (c) The complex of the expected oligomeric state and varying numbers of bound MCL-1 protein(s) was modelled with AlphaFold2 with the average pAE scores for the peptide residues shown (only the best model was used and lower values in blue indicate a better model);

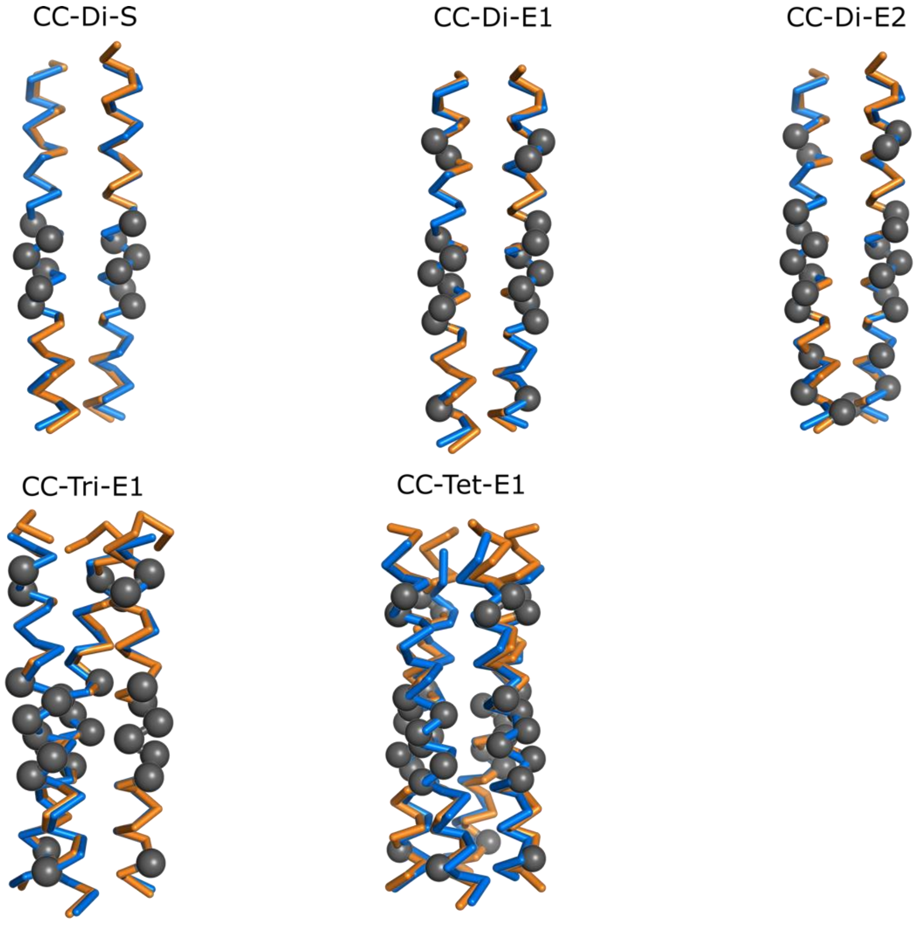


**Figure S3. Alignment of AlphaFold2 models of binder designs with original peptides.** The binders are shown as orange Ca ribbons with Ca of grafted hotspot residues shown as grey spheres. They were aligned to the original peptide crystal structures, shown as a blue Ca ribbon. CC-Di_S, CC-Di_E1 and CC-Di_E2 were aligned to CC-Di (PDB: ‘(PDB: 4DZM), CC-Tri_E1 was aligned to CC-Tri (PDB: 4DZK), CC-Tet_E1 was aligned to CC-Tet (PDB: 3R4A).

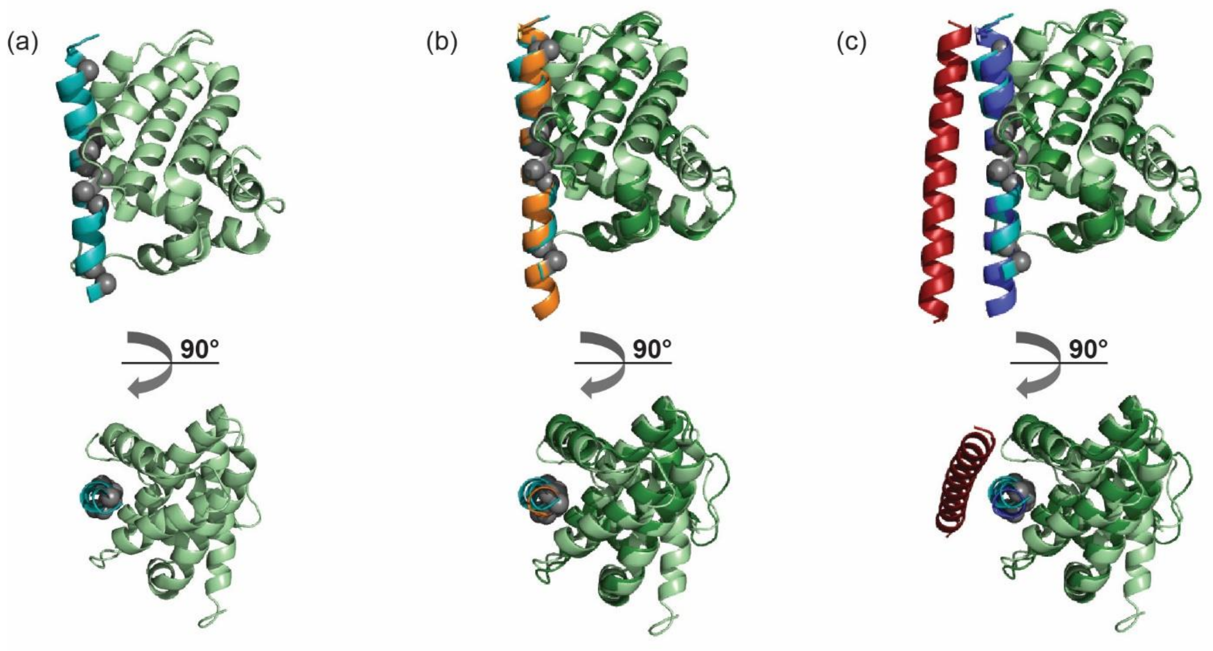


**Figure S4. AlphaFold2 models of 1:1 complexes:** (a) Side (above) and top down (N-terminus down to C-terminus, below) view of the NOXA-B/MCL-1 complex (PDB 2JM6) with NOXA-B in cyan and MCL-1 in light green; (b) Model of Mono_E1/MCL-1 complex viewed from the side (above) and top down (below, with Mono_E1 shown in orange and hot-spot residues shown as gray spheres in association with MCL-1 in dark green, overlaid with NOXA-B/MCL-1 from 2JM6, with the NOXA-B peptide in cyan and MCL-1 in light green; (c) Model of CC-Di-A:CC-Di-B_S:MCL-1 (with CC-Di-A in red, CC-Di-B_S in dark blue, hot-spot residues as gray spheres and MCL-1 in dark green, overlaid with NOXA-B:MCL-1 from PDB ID 2JM6, with the NOXA-B peptide in cyan and MCL-1 in light green).

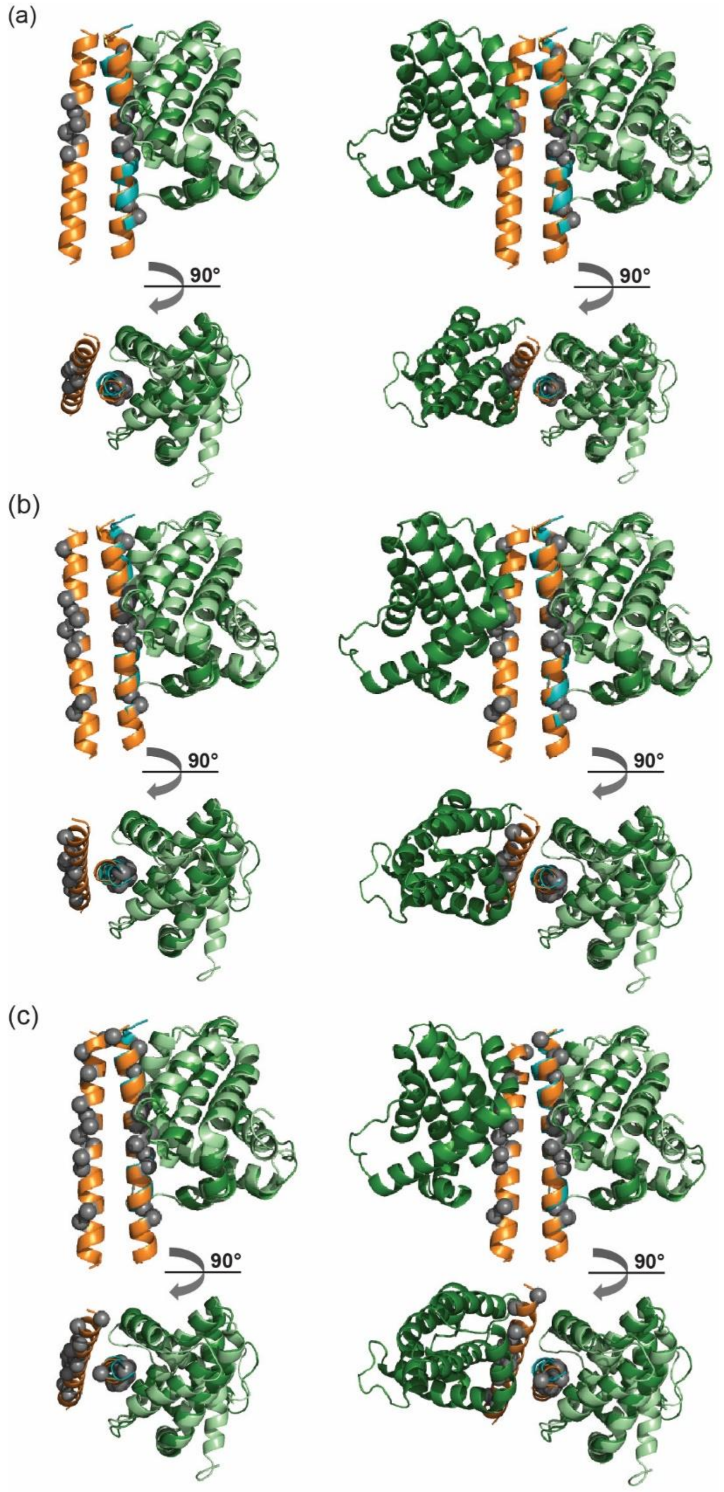


**Figure S5. AlphaFold2 models of dimeric peptide sequences in complex with MCL-1.** Models showing: (a) 2:1 (left) and 2:2 (right) CC-Di_S/MCL-1 complexes; (b) 2:1 (left) and 2:2 (right) CC-Di_E1/MCL-1 complexes; and, (c) 2:1 (left) and 2:2 (right) CC-Di_E2/MCL-1 complexes, viewed from the side (above) and top down (N- to C-terminus, below). In all models, peptide chains are shown in orange with hot-spot residues shown by gray spheres, and MCL-1 in dark green. These structures are overlaid with NOXA-B/MCL-1 (from PDB ID 2JM6, with NOXA-B in cyan and MCL-1 in light green).

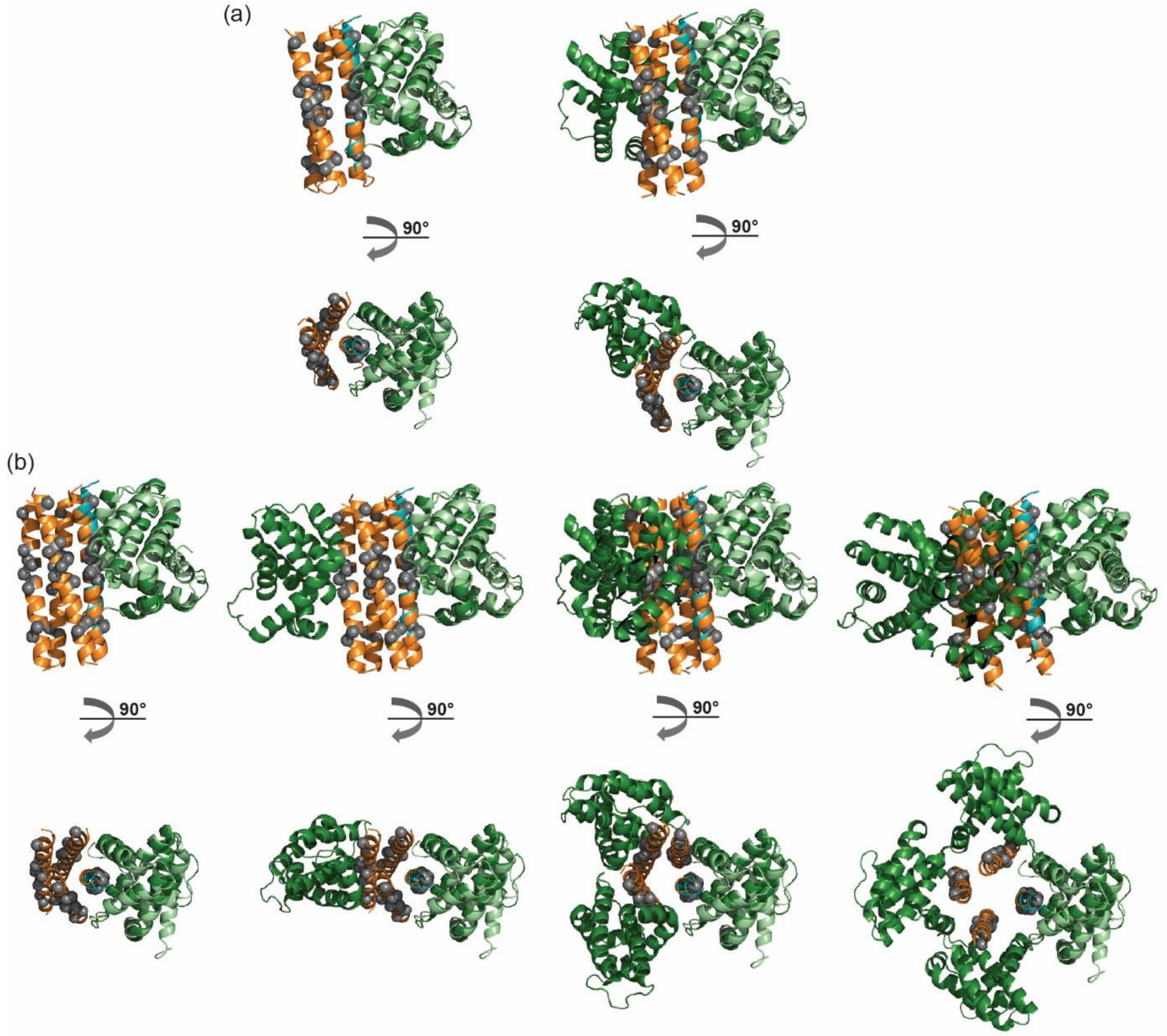


**Figure S6. AlphaFold2 models of trimeric and tetrameric peptide sequences in complex with MCL-1.** Models showing: (a) left to right 3:1 and 3:2 CC-Tri_E1/MCL-1 complexes viewed from the side (above) and top down (N- to C-terminus, below); (b) left to right 4:1, 4:2, 4:3 and 4:4 CC-Tet_E1/MCL-1 complexes viewed from the side (above) and top down (N- to C-terminus, below). In all models, peptide chains are shown in orange with hot-spot residues shown by gray spheres, and MCL-1 in dark green. These structures are overlaid with NOXA-B/MCL-1 (from PDB ID 2JM6, with NOXA-B in cyan and MCL-1 in light green). The 3:3 CC-Tri_E1/MCL-1 structure has been omitted, as the peptide no longer assembles as a coiled-coil scaffold in the model.

| | 1 | 2 | 3 | 4 | Total |
|---|---|---|---|---|---|
| **NOXA-B** | **908.8** | | | | **908.8** |
| **Mono_E1** | **884.9** | | | | **884.9** |
| **CC-Di-A:CC-Di-B_S** | **568.2** | | | | **568.2** |
| **CC-Di_S** | **524.8** | **526.2** | | | **1050.9** |
| **CC-Di_E1** | **793.5** | **792.7** | | | **1586.2** |
| **CC-Di_E2** | **1162.62** | **1175.6** | | | **2338.2** |
| **CC-Tri_E1** | **770.9** | **765.4** | **779.5** | | **2315.8** |
| **CC-Tet_E1** | **662.1** | **756.2** | **621.0** | **739.2** | **2738.5** |

**Table S1. Calculated solvent-exposed surface area of each scaffold's grafted hot-spot residues per chain and in total for the entire scaffold**. Values shown are in Å$^2$. Values were calculated from the first model in 2JM6 for NOXA-B, or the models shown in Fig. S1. Values were calculated in PyMol 3.0.

| | NOXA-B | Mono_E1 | CC-Di-A:CC-DiB_S | CC-Di_S | | CC-Di_E1 | | CC-Di_E2 | | CC-Tri_E1 | | | CC-Tet_E1 | | | |
|---|---|---|---|---|---|---|---|---|---|---|---|---|---|---|---|---|
| **Chain** | 1 | 1 | 1 | 1 | 2 | 1 | 2 | 1 | 2 | 1 | 2 | 3 | 1 | 2 | 3 | 4 |
| Lys2 | | | | | | | | 89 | 89 | | | | | | | |
| Leu4 | 58 | 61 | | | | 55 | 57 | 60 | 70 | 57 | 60 | 59 | 45 | 56 | 45 | 57 |
| Glu7 | | | | | | | | 56 | 58 | | | | | | | |
| Leu11 | 73 | 71 | 71 | 72 | 71 | 72 | 70 | 73 | 58 | 65 | 66 | 66 | 43 | 65 | 43 | 66 |
| Arg12 | 65 | 54 | 64 | 57 | 59 | 57 | 58 | 72 | 50 | 59 | 56 | 60 | 68 | 57 | 65 | 56 |
| Ile14 | 48 | 60 | 41 | 40 | 40 | 40 | 40 | 42 | 50 | 33 | 37 | 36 | 8 | 45 | 13 | 34 |
| Gly15 | 42 | 44 | 46 | 44 | 44 | 45 | 44 | 44 | 41 | 51 | 47 | 48 | 47 | 47 | 45 | 49 |
| Asp16 | 41 | 60 | 53 | 40 | 43 | 38 | 41 | 39 | 46 | 33 | 28 | 34 | 29 | 26 | 27 | 29 |
| Val18 | 49 | 62 | 62 | 60 | 60 | 60 | 61 | 61 | 40 | 62 | 61 | 61 | 55 | 62 | 56 | 61 |
| Asn19 | | | | | | | | 65 | 62 | | | | | | | |
| Leu25 | 64 | 54 | | | | 52 | 52 | 50 | 49 | 48 | 48 | 47 | 32 | 51 | 30 | 52 |
| Asn26 | 79 | 60 | | | | 52 | 51 | 53 | 53 | 51 | 53 | 50 | 39 | 36 | 40 | 32 |

**Table S2. Calculated solvent-exposed surface area per residue for each scaffold's grafted hot spots, by chain**. Values shown are percent solvent exposure. Values were calculated from the first model in 2JM6 for NOXA-B, or the models shown in Fig. S1. Values were calculated in PyMol 3.0.

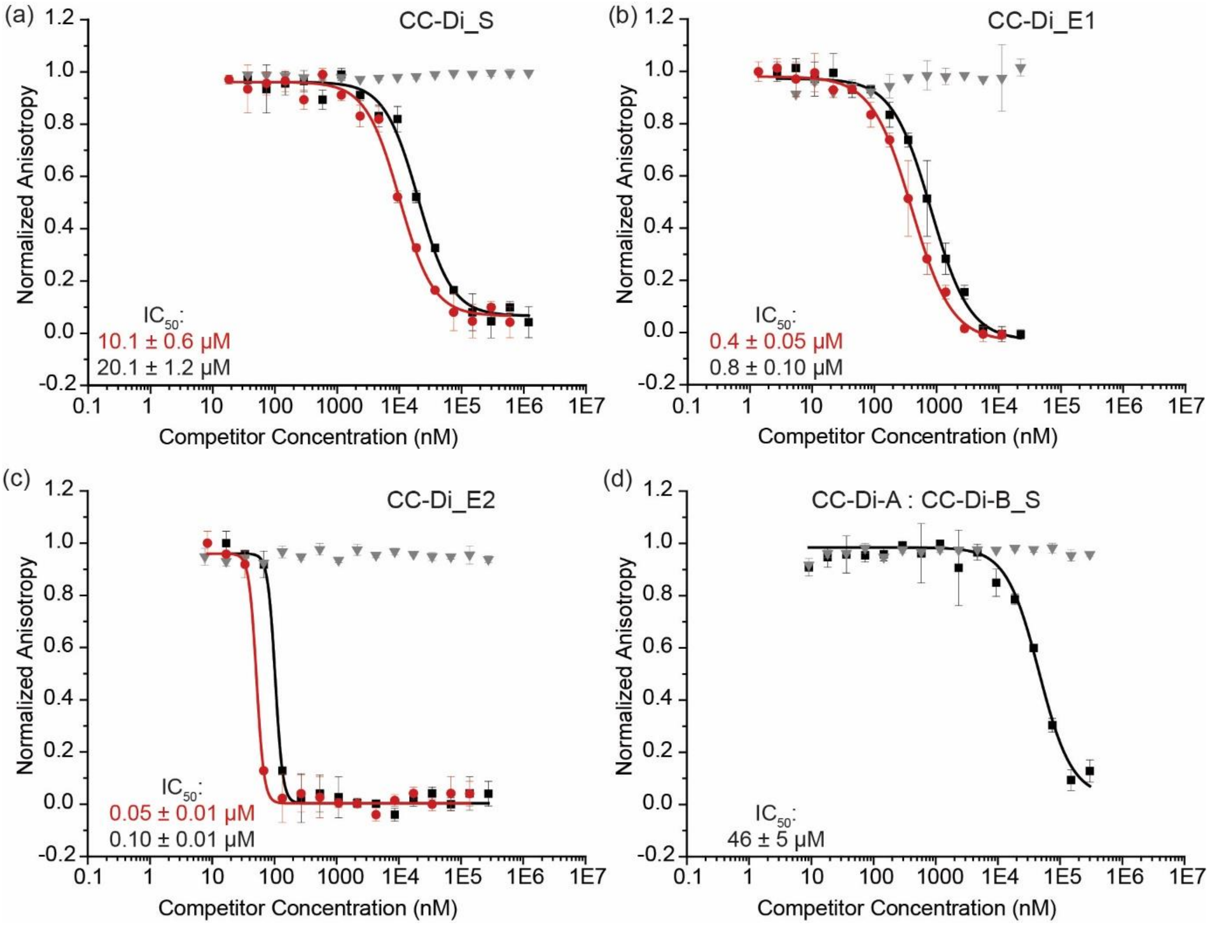


**Figure S7**. **Fluorescence anisotropy competition assays of peptides.** Traces show the peptide constructs disrupting the FAM-Ahx-BID / MCL-1 complex (black squares), and against FAM-Ahx-BID/BCL-$x_L$ (gray triangles). Black squares show the competitor concentration normalized to the total peptide concentration (i.e. total number of grafted binding sites, to show binding relative to peptide concentration); red squares show the same data with the concentration normalized per scaffold's designed oligomerization state (to show binding relative to scaffold concentration). Calculated $IC_{50}$ values for each experiment are shown in the panels. Conditions: 150 nM protein, 25 nM FAM-Ahx-BID, 20 °C, Tris buffer (50 mM Tris-HCl, 150 mM NaCl, pH 7.4, error bars from n = 3).

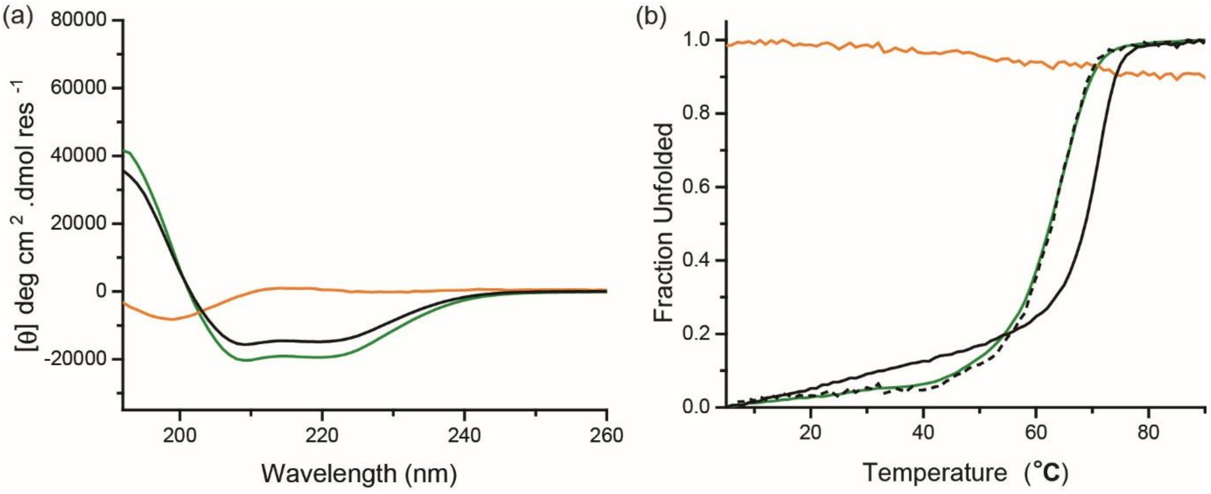


**Figure S8**. **CD analysis of NOXA-B, MCL-1 and the NOXA-B/MCL-1 complex**. Panel (a) shows the overlaid wavelength scans of the peptide NOXA-B (orange), the protein MCL-1 (green) and the NOXA-B/MCL-1 complex (black) at 20 °C. The NOXA-B peptide alone shows a single minimum at 199 nm characteristic of an unfolded structure, and the MCL-1 protein and NOXA-B/MCL-1 peptide-protein complex show the double minima at 208 and 222 nm characteristic of alpha-helical structure. Panel (b) shows a thermal unfolding experiment monitoring the signal at 222 nm from 5 – 85 °C for NOXA-B (orange), MCL-1 (green) and the NOXA-B/MCL-1 complex (solid black line). A theoretical unfolding spectra for the NOXA-B/MCL-1 complex (dashed black line) was generated by averaging the NOXA-B and MCL-1 unfolding curves. Conditions: 25 µM NOXA-B, 25 µM MCL-1, 25:25 µM NOXA-B/MCL-1 in 20 mM phosphate, 100 mM NaCl, pH 7.4.

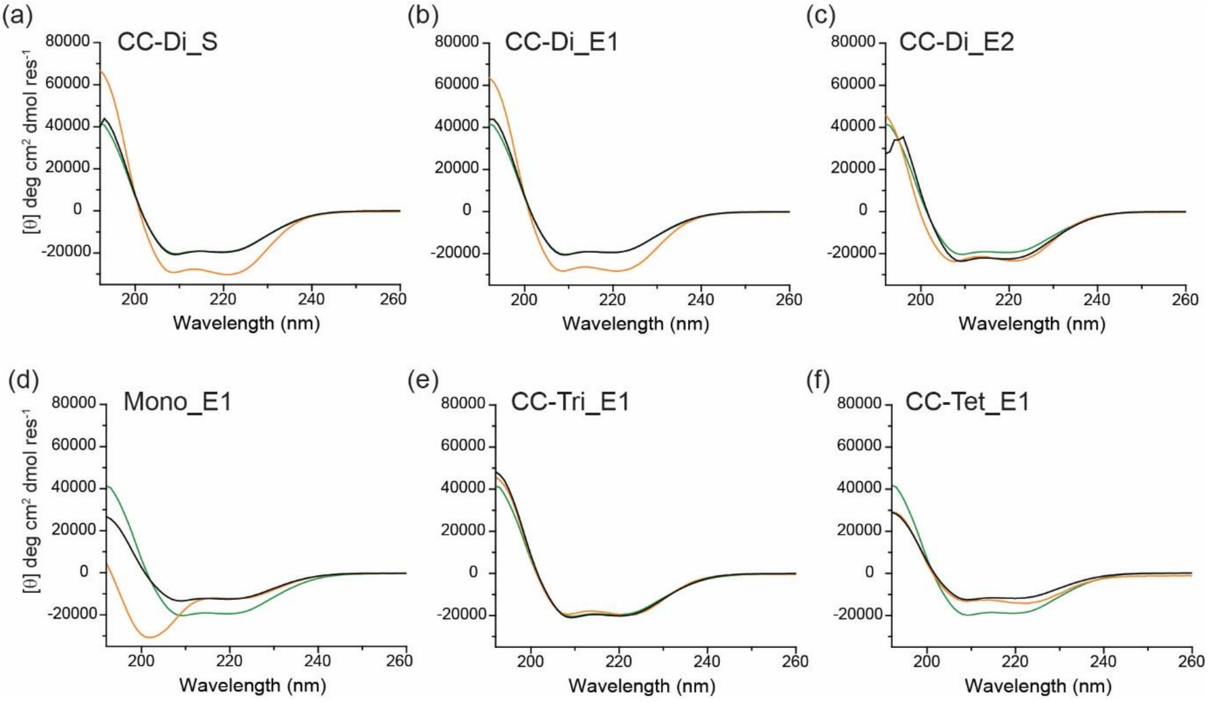


**Figure S9, CD spectra for coiled-coil peptides, the protein MCL-1 and peptide/protein complexes:** (a) CC-Di_S; (b) CC-Di_E1; (c) CC-Di_E2; (d) Mono_E1; (e) CC-Tri_E1; (f) CC-Tet_E1 (peptide samples are shown in orange, MCL-1 in green and peptide/MCL-1 complexes in black; concentration = 25 µM of each component in 20 mM phosphate, 100 mM NaCl, pH 7.4, wavelength spectra acquired at 20 °C).

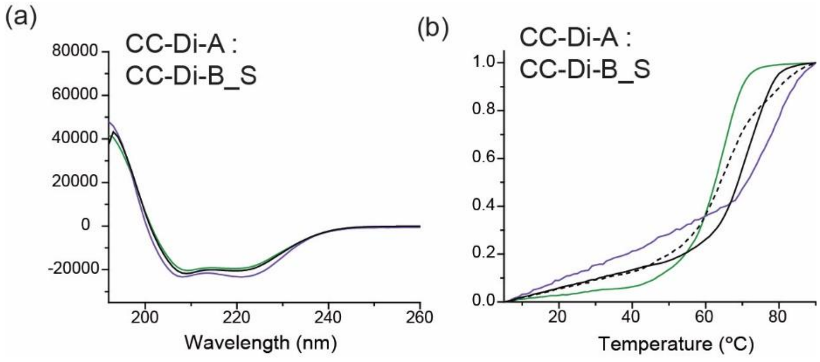


**Figure S10. CD analyses for CC-Di-A:CC-Di-B_S, MCL-1 and CC-Di-A/CC-Di-B_S/MCL-1:** (a) spectra (at 20 °C); and, (b) thermal unfolding curves (heterodimer peptide pair in purple, MCL-1 in green and peptide/MCL-1 complexes in black, theoretical unfolding spectra of complexes in black dashes, concentration = 25 µM of each component in 20 mM phosphate, 100 mM NaCl, pH 7.4).

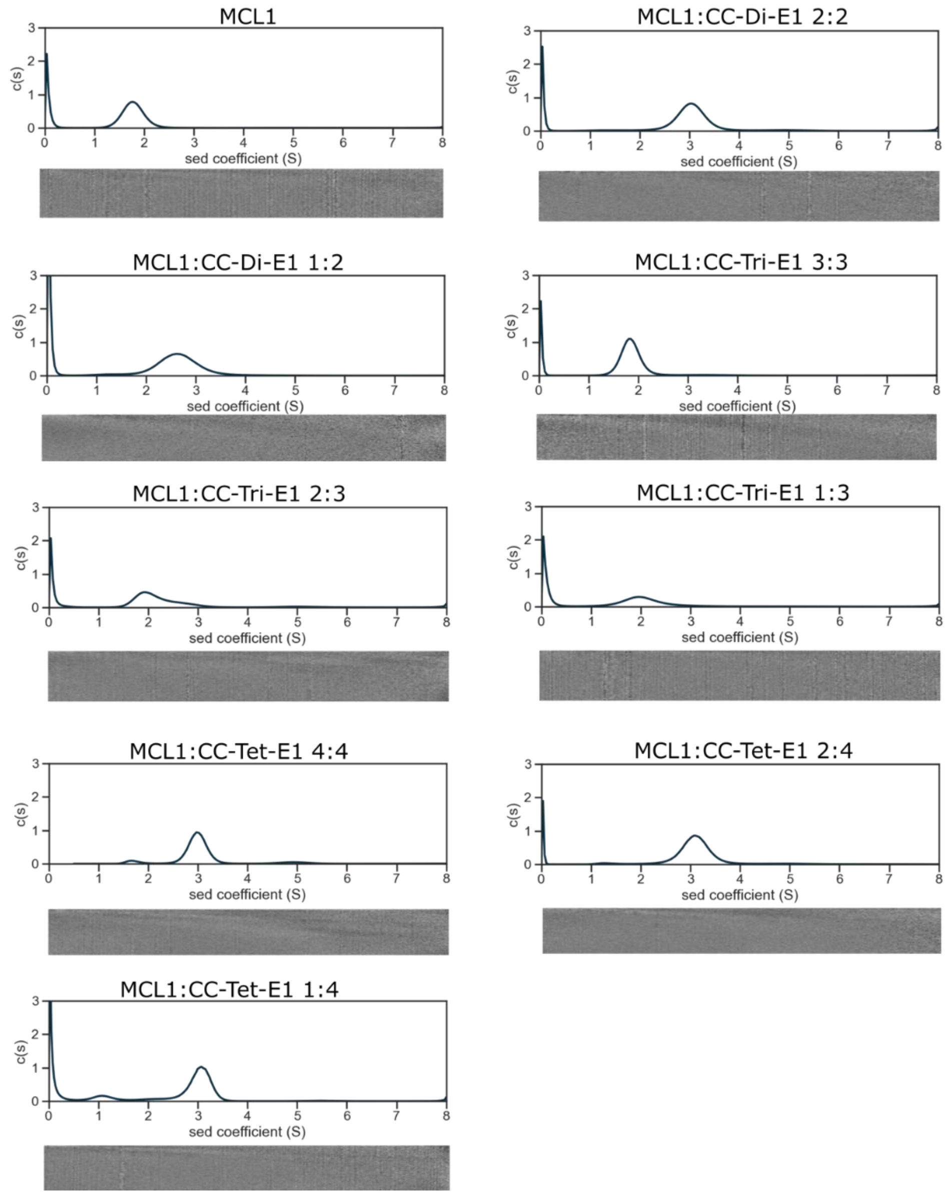


**Figure S11. Sedimentation velocity (SV) analytical ultracentrifugation (AUC) of MCL-1 and peptide mixtures.** The data was fitted as a continuous distribution using SEDFIT. Residuals of the fits are shown as a bitmap below the respective distribution. Greyscale shade indicates difference between the model and raw data over the radial range of the fit (residuals <-0.05 black, > 0.05 white). The main peak was integrated, and the corresponding values are shown in table S3. Conditions: 30 µM MCL-1 and peptide concentration ranging from 30 µM to 120 µM depending on the ratio, 20 °C, Tris buffer (50 mM Tris, 150 mM NaCl, pH 7.4).

**Table S3. Molecular Weight Data Determined from AUC Experiments**

| Protein and peptide | Ratio | Sedimentation coefficient (S) | Peak area (% of total) | Molecular weight with best fit (kDa) | Suggested complex | Theoretical molecular weight suggested complex (kDa) |
|---|---|---|---|---|---|---|
| MCL1 | 1 | 1.8 ± 0.2 | 70 | 19.2 ± 0.7 | MCL1 | 17.735 |
| MCL1:CC-Di_E1 | 2:2 | 3.0 ± 0.3 | 75 | 39.9± 1.3 | MCL1:CC-Di-E1 2:2 | 42.654 |
| | 1:2 | 2.6 ± 0.4 | 59 | 30.8 ± 1.9 | MCL1:CC-Di-E1 1:2 | 24.919 |
| MCL1:CC-Tri_E1 | 3:3 | 1.9 ± 0.2 | 78 | 20.2 ± 0.7 | MCL1:CC-Tri-E1 1:1 | 21.325 |
| | 2:3 | 2.2 ± 0.4 | 65 | 26 ± 2 | MCL1:CC-Tri-E1 1:3 | 28.505 |
| | 1:3 | 2.0 ± 0.5 | 53 | 25 ± 3 | MCL1:CC-Tri-E1 1:3 | 28.505 |
| MCL1:CC-Tet_E1 | 4:4 | 3.0 ± 0.2 | 82 | 33.8 ± 0.6 | MCL1:CC-Tet-E1 1:4 | 32.087 |
| | 2:4 | 3.1 ± 0.3 | 81 | 39.8 ± 1.2 | MCL1:CC-Tet-E1 1:4 | 32.087 |
| | 1:4 | 3.0 ± 0.2 | 55 | 27.1 ± 0.5 | MCL1:CC-Tet-E1 1:4 | 32.087 |

Conditions as for Fig. S11.

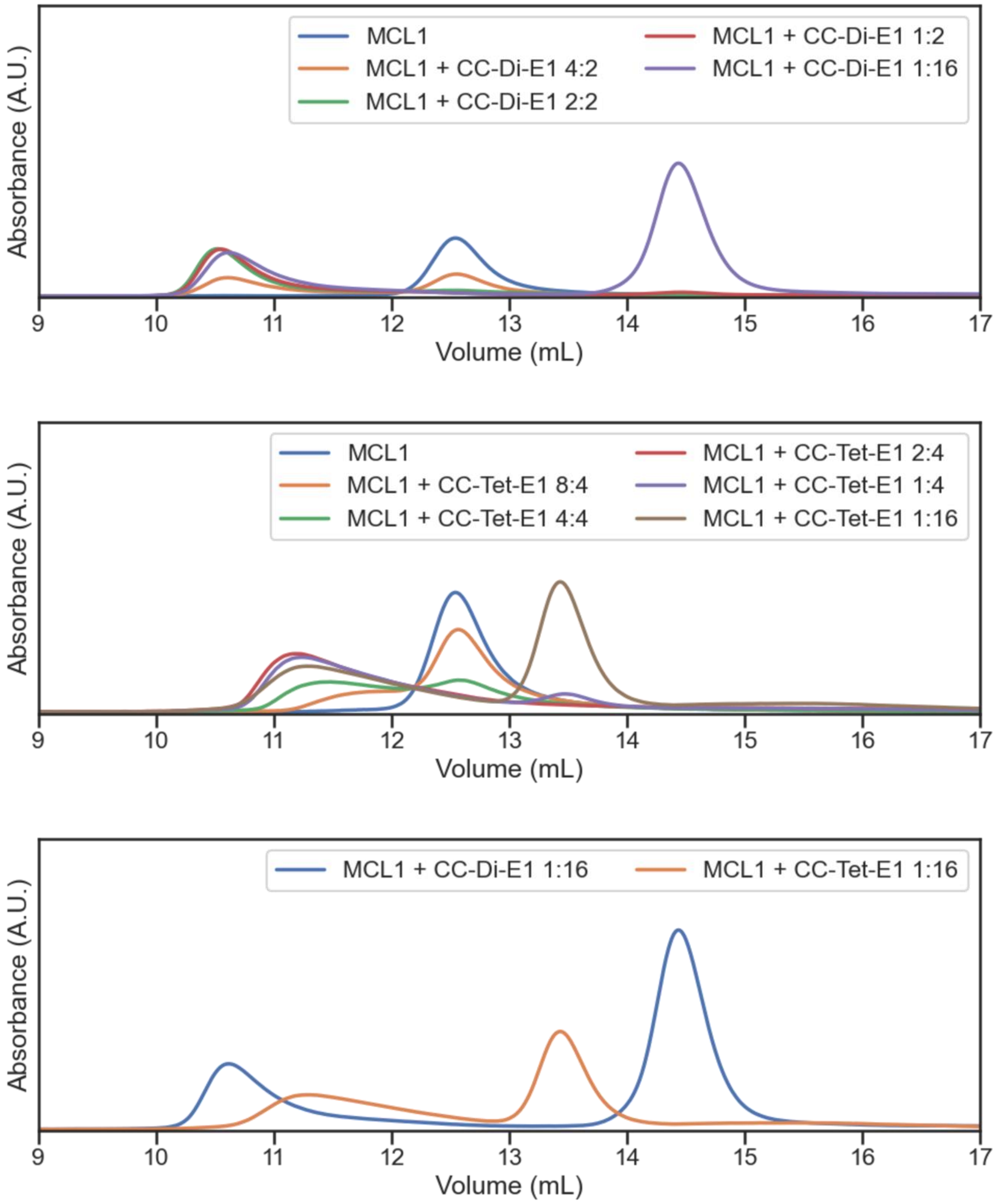


**Figure S12. Size exclusion chromatography (SEC) of MCL-1 and peptide mixtures.** A fixed concentration of MCL-1 (30 µM) was mixed with various concentrations of peptide binders. 20 µL sample was injected on a superdex™ 75 increase 10/300 (Cytiva), UV light absorbance was followed at 230 nm and is shown as raw data after subtraction of buffer absorbance. Conditions: 30 µM MCL-1 and peptide concentrations ranging from 15 µM to 480 µM, 20 °C, Tris buffer (20 mM Tris, 150 mM NaCl, pH 7.5). For CC-Di_E1, the formation of a peak corresponding to the 2:2 complex was observed. The unbound MCL-1 peak disappeared completely at the equimolar ratio indicating that the preferred binding complex is 2:2. No peak at a 1:2 MCL-1 – CC-Di_E1 complex was observed even at a 16-fold excess of peptide, highlighting the highly cooperative binding observed in CD melting experiments.

For CC-Tri_E1 no binding was observed under these conditions possibly due to the weaker MCL-1 affinity. For CC-Tet_E1, a very broad peak sloped towards smaller molecular weight species was observed. The sloped nature could be explained by dissociation on the column due to the modest affinity. Therefore, the location of the peak may not correspond to the starting complex. The monomeric MCL-1 peak disappeared at the 2:4 ratio suggesting the initial complex formed may have this ratio. While the MCL-1:peptide complex peak initially shifted towards higher MW species with increasing peptide concentration, the 1:16 ratio showed a clear shift towards lower MW, potentially indicating the prevalence of 1:4 complexes.

### AlphaFold2 Modelling

The AlphaFold2[1] models were created using localcolabfold[2] on a Nvidia RTX A5500. For the oligomeric state predictions of the peptides no MSA was used the number of recycles was limited to three and the model weights of alphafold2_multimer_v3[3] were used. The complexes with MCL-1 were run with the same model but using twelve recycles, MSA and templates to easy prediction of the MCL-1, apart from NOXA-B no template were found for the peptides. pLDDT and pAE averages were calculated for the peptide residues for the best model and for all models combined using a python script. For overlays of AlphaFold2 models with NOXA-B/MCL-1 throughout the main text and supporting information, the first NOXA-B/MCL-1 model in the PDB 2JM6 was used for comparison.

### Protein Overexpression and Purification

Plasmids for MCL-1 (pET28a vector, kanamycin resistance, residues 172 – 327, fusion protein $M_W$ = 37,337, fusion protein extinction coefficient = 24,075 $M^{-1}$ $cm^{-1}$, cleaved protein $M_W$ = 17,735 cleaved protein extinction coefficient = 19,480 $M^{-1}$ $cm^{-1}$) and BCL-$x_L$ (pET28a vector, kanamycin resistance, residues 1 – 198 missing residues 26 – 81, fusion protein $M_W$ = 30,763, fusion protein extinction coefficient = 41,035 $M^{-1}$ $cm^{-1}$, cleaved protein $M_W$ = 17,489, cleaved protein extinction coefficient = 36,440 $M^{-1}$ $cm^{-1}$) were transformed into the BL21-Gold *E. coli* strain, plated on LB-agar kanamycin selection plates, and single colonies used to make glycerol stocks. These stocks were used inoculate small overnight starter cultures (1:100 dilution into expression media, all media as LB broth containing 50 µg/mL kanamycin). Expression media was incubated at 37 °C until $OD_{600}$ reached 0.6; the temperature was then lowered to 18 °C and 250 µM IPTG added, and the protein overexpressed overnight. The next day, cells were pelleted (30 min, 5,000 rpm, Beckman Coulter JXN-26 floor centrifuge, fixed-angle JLA-8.100 rotor), and collected and frozen overnight at -80 °C to improve lysis. Cells were thawed and re-suspended in lysis buffer (25 mM Tris, 500 mM NaCl, pH 8.0; generally 25 mL buffer per 1 L of culture) containing protease inhibitor (1 tablet per 6-8 L cell pellet, cOmplete, Mini, EDTA-free protease inhibitor cocktail, Roche), lysozyme (5 mg per 6-8 L cell pellet, Roche) and DNAse (3 mg per 6-8 L cell pellet, Roche) and the mixture stirred for 20 min before sonication in a salt/ice bath (Q500 sonicator with a 12 mm probe, QSonica, cycles of 4 sec on 8 sec off, 60% amplitude, 6 min total). The lysate was clarified via centrifugation (45 min, 15,000 rpm, Beckman Coulter JXN-26 floor centrifuge, fixed-angle JA-25.50 rotor), passed through a 0.2 µm filter and applied to a 5 mL His-trap HP column (Merck) pre-equilibrated with wash 1 buffer (10 mM imidazole, 25 mM Tris, 500 mM NaCl, pH 8.0). The column was then washed with wash 2 buffer (10 column volumes, 20 mM imidazole, 25 mM Tris, 500 mM NaCl, pH 8.0), wash 3 buffer (10 column volumes, 40 mM imidazole, 25 mM Tris, 500 mM NaCl, pH 8.0) and wash 4 buffer (10 column volumes, 100 mM imidazole, 25 mM Tris, 500 mM NaCl, pH 8.0) before elution (10 column volumes elution buffer, 400 mM imidazole, 25 mM Tris, 500 mM NaCl, pH 8.0). An SDS-PAGE gel (4-20% gradient gel, Bio-Rad Laboratories) confirmed the fractions containing the purified fusion protein, which were

then pooled for overnight dialysis (4 L total volume, 20 mM Tris, 150 mM NaCl, pH 8.0, MWCO 10,000 Da dialysis tubing, Thermo Scientific) and cleavage of the N-terminal His-SUMO affinity tag via His-Ulp1 SUMO protease (1:20 ratio of protease:fusion protein). The next day the contents of the dialysis tubing were passed through a 0.2 μm filter before application to a 5 mL His-trap HP column. The flow through, containing the purified and Ulp1-cleaved protein, was collected, while uncleaved fusion protein, N-terminal His-SUMO affinity tag and His-Ulp1 remained bound to the nickel column. After confirmation via SDS-PAGE, the cleaved and purified protein was concentrated (10,000 MWCO Thermo Scientific) for size exclusion chromatography (HiLoad[TM] 26/60 Superdex 75 column, GE healthcare, Akta Prime Plus, 2 mL / min flow rate, 20 mM Tris, 250 mM NaCl, 0.5 mM DTT, 2.5% glycerol, pH 8.0), the fractions containing the purified protein collected, and the protein concentrated (generally 150 – 250 μM) for storage in at -80 °C. Before use in experiments, concentrations were calculated using the absorbance at A280 on a NanoDrop One (ThermoScientific).

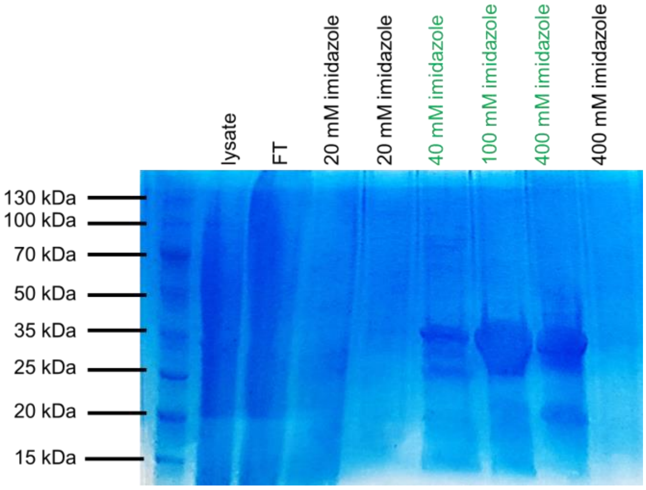


**Purification of His$_6$-SUMO-MCL-1**. SDS-PAGE gel of forward nickel column fractions for the purification of affinity tagged MCL-1. Green fractions were cleaved with Ulp1 SUMO protease, then re-applied to the nickel column.

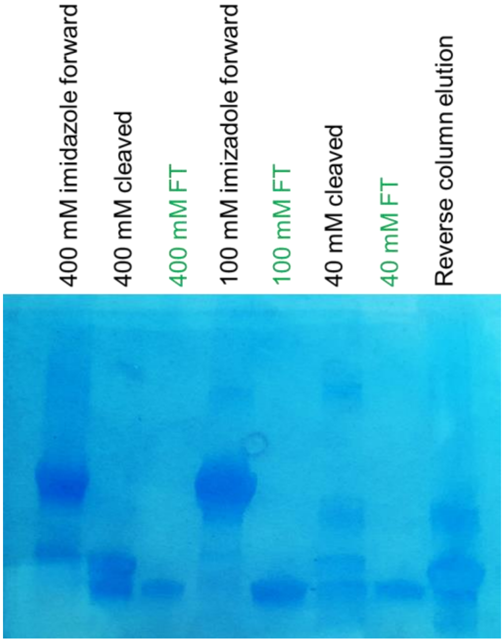

**Purification of MCL-1 after affinity tag cleavage**. SDS-PAGE gel of reverse nickel column fractions for the purification of MCL-1 after the removal of the affinity tag. Green fractions were combined then concentrated together for size exclusion chromatography purification.

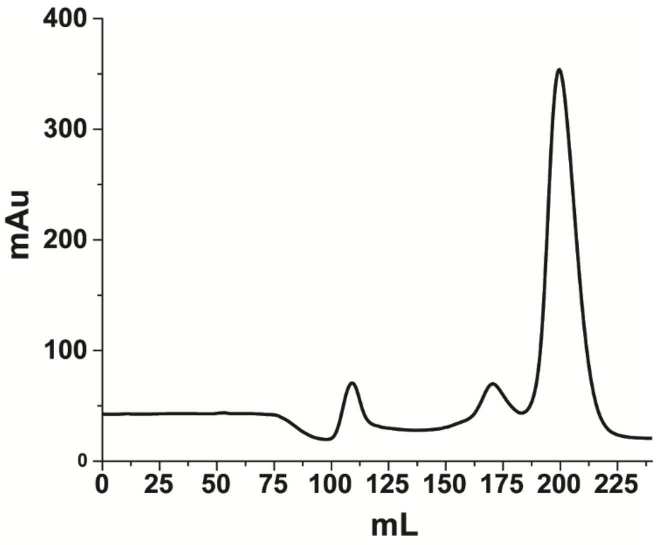


**Size exclusion chromatography run of MCL-1 after affinity tag cleavage and the reverse nickel column**. Fractions of pure protein were collected from 195 – 225 mL.

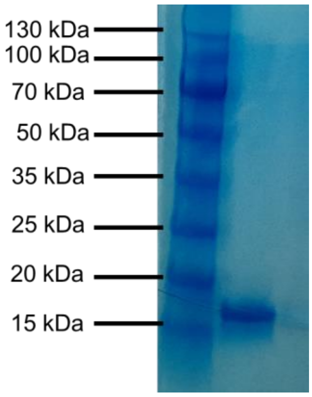


**SDS-PAGE gel of MCL-1 after size exclusion chromatography**. Band represents combined pure protein fractions collected between 195 – 225 mL in from the above SEC trace.

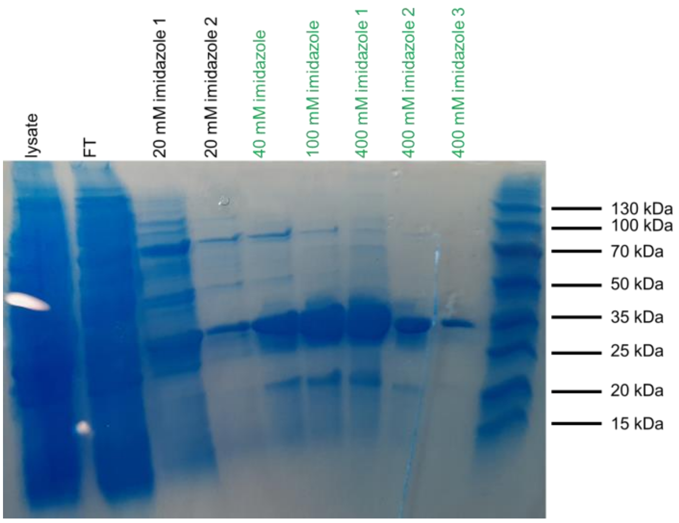


**Purification of His$_6$-SUMO-BCL-x$_L$**. SDS-PAGE gel of forward nickel column fractions for the purification of affinity tagged BCL-x$_L$. Green fractions were cleaved with Ulp1 SUMO protease, then re-applied to the nickel column.

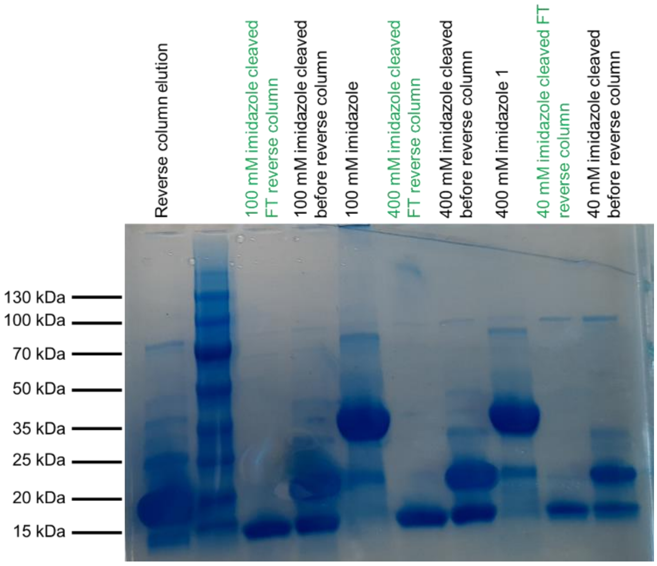


**Purification of BCL-$_{xL}$ after affinity tag cleavage**. SDS-PAGE gel of reverse nickel column fractions for the purification of BCL-x$_L$ after the removal of the affinity tag. Green fractions were combined then concentrated together for size exclusion chromatography purification.

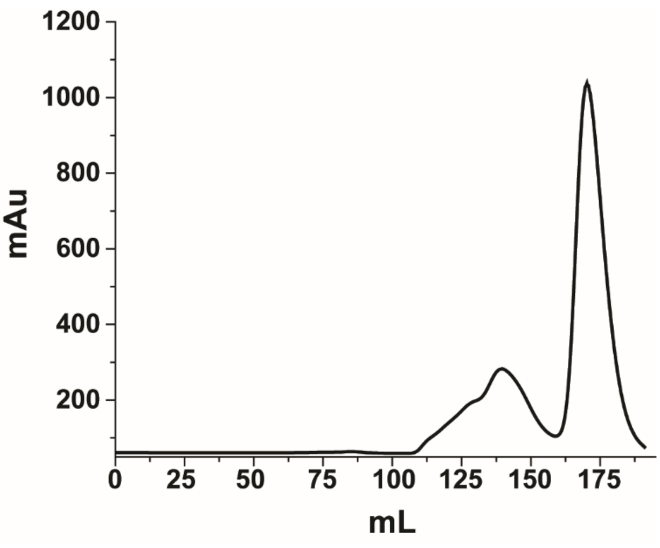


**Size exclusion chromatography run of BCL-x$_L$ after affinity tag cleavage and the reverse nickel column**. Fractions of pure protein were collected from 165 – 190 mL.

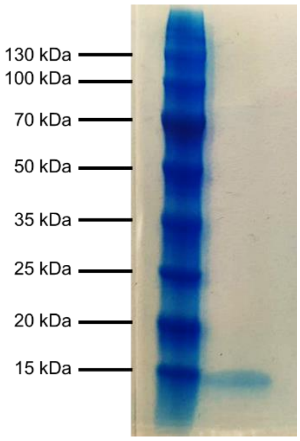


**SDS-PAGE gel of BCL-x$_L$ after size exclusion chromatography**. Band represents combined pure protein fractions collected between 165 – 190 mL in from the above SEC trace..

### Fluorescence Anisotropy Competition Assays

Competition assays were performed in 20 mM Tris, 150 mM NaCl, pH 7.4 in low volume 384 well plates (Greiner Bio-One). Protein concentrations were fixed at 150 nM and the tracer FAM-Ahx-BID fixed at 25 nM. For all plates, a control experiment with either MCL-1/FAM-Ahx-BID or BCL-x$_L$/FAM-Ahx-BID was titrated against a BID wild-type sequence and used to optimize plate height and gain. All experiments were performed in triplicate (for both the test wells with fluorescent tracer peptide and control wells lacking the fluorescent tracer), and plates were read after 1 h and 24 h incubation at room temperature. Data shown throughout was collected at 24 h. Data was collected with a CALRIOstar plate reader (BMG Labtech), and excitation at 482 nm (bandwidth 16 nm) and emission at 530 nm (bandwidth 40 nm).

Perpendicular (P) and parallel (S) intensities of the control wells were averaged and deducted from each corresponding samples to give the corrected values, $P_{corr}$ ($P_{corr} = P_{sample} – P_{av.\ control}$) and $S_{corr}$ ($S_{corr} = S_{sample} – S_{av.\ control}$). The total sample intensities (I) and anisotropies (r) were calculated with the follow equations:

$$I = (2 \times P_{corr}) + S_{corr}$$

$$r = (S_{corr} – P_{corr}) / I$$

Fluorescence anisotropy values were plotted against peptide competitor concentrations and fit to a logistic function of the form $Y = A2 + (A1 – A2) / (1 + (x/x_0)^p)$ to determine the half-maximal inhibitory concentration ($IC_{50}$) of the peptide competitor (where A2 is the upper asymptote and A1 the lower asymptote).

### Circular Dichroism

Stock solutions of protein (stored in the SEC buffer) or peptide (peptide stocks generally prepared at 200 – 500 µM in deionized water from solid peptides) were diluted into phosphate buffer (20 mM phosphate, 100 mM NaCl, pH 7.4) to a final concentration of 25 µM for individual components, and at a 1:1 ratio (final concentration of all components set as 50 µM for homomeric species and 75 µM for heterodimeric complex with MCL-1, or 1:1:1 ratio) of the number of binding sites on the coiled-coil peptides to BH3 binding domain on the protein. For example dimeric coiled-coils CC-Di_S, CC-Di_E1 and CC-Di_E2 presenting 2 grafted binding sites were prepared in a 2:2 ratio to MCL-1, which contains a single BH3 binding domain; the coiled-coil heterodimer, which contains a single grafted site was prepared in the ratio 1:1:1 of CC-Di_A:CC-Di_B_S:MCL-1, the trimeric CC-Tri_E1 prepared in a 3:3 ratio with MCL-1 and so on. Wavelength spectra were collected (average of 5 scans, from 190 – 300 nm) to evaluate secondary structure of the individual components and the peptide:protein complexes at 20 °C. Thermal unfolding experiments monitored the α helical minima at 222 nm from 5 – 85 °C with a heating ramp of 20 °C/h, and $T_m$ was calculated from the mid-point of unfolding transitions. A background spectrum of the buffer alone was also collected and subtracted from raw sample ellipticity values during data conversion.

Data were collected on an APP Chirascan CD spectropolarimeter using 1 mm pathlength quartz cuvettes. Raw ellipticity data were converted to mean residue ellipticity (MRE) using the following equation:

$$MRE = (([\theta]_\lambda – [\theta]_0) \times M_w) / (n \times l \times c)$$

Where $[\theta]_\lambda$ is the observed ellipticity at a wavelength λ in mdeg, $[\theta]_0$ is the ellipticity observed for the matched buffer, $M_w$ = molecular weight (g $mol^{-1}$), $n$ = number of amide bonds, $c$ = sample concentration (mg $mL^{-1}$); $l$ = pathlength of the cuvette in cm. For thermal unfolding curves, the fraction folded was calculated with the following equation

$$\theta = [\ \theta_T – \theta_{unfolded}\ ] / [\ [\theta_{folded} – \theta_T] + \theta_T - \theta_{unfolded}]$$

where $\theta_T$ represents the ellipticity at a given temperature, $\theta_{unfolded}$ the ellipticity for the fully unfolded species, and $\theta_{folded}$ for the fully folded species.

### Native Mass Spectrometry Analysis

Peptides were dissolved in either HPLC grade water or 100mM ammonium acetate pH 6.8 and dialysed overnight against a 500 Da MWCO membrane. The peptides were then diluted to stock solution of 100 µM in 100 mM ammonium acetate pH 6.8, verified via nanodrop measurement of total absorbance at 280 nm. MCL-1 was exchanged into 100 mM ammonium acetate pH 6.8 using a 10 kDa Amicon Ultra 0.5 mL centrifugal concentrator. Both the peptides and MCL-1 were stored at -20 °C prior to use.

For native mass spectrometry analysis, a protein:peptide ratio of 1:1 was used (5 µM protein: 5 µM peptide). Native mass spectrometry was performed on a QExactive HF mass spectrometer (Thermo Fisher Scientific) equipped with a nanoelectrospray ionisation source. Nanoelectrospray was performed using gold-coated borosilicate glass capillaries, pulled in-house. Positive ionisation mode was used throughout with the capillary voltage set to 1.4 kV. The source temperature was set at 250 ºC, in source dissociation at 0, S-lens RF at 100, max. injection time 50 ms, AGC target $5e^{6}$. The resolution in the Orbitrap was set to 15,000. Mass spectra were acquired over 1000-6000 *m/z* range. All data was analysed using XCalibur (*v*.4.2). A minimum of three charge states was used in all cases to confirm the molecular weights of the detected complexes.

**Theoretical and measured masses of all peptides and protein complexes detected by native MS.** The error reported is the standard deviation measured between charge states. The mono-isotopic masses and average isotopic masses are reported for peptides and proteins/protein complexes, respectively.

| Peptides/MCL-1 Complexes | Theoretical mass (Da) | Measured mass (Da) |
|---|---|---|
| CC-Di_S | 3462.96 | 3462.99 |
| CC-Di_E1 | 3590.06 | 3590.07 |
| CC-Di_E2 | 3633.06 | 3633.12 |
| CC-Tri_E1 | 3590.06 | 3590.07 |
| CC-Tet_E1 | 3589.10 | 3589.14 |
| CC-Di-A | 3411.70 | 3411.75 |
| CC-Di-B_S | 3460.11 | 3460.14 |
| MCL-1 | 17735.0 | 17735.8 ± 0.5 |
| MCL-1:CC-Di_S | 21200.1 | 21201.2 ± 0.6 |
| 2x(MCL-1:CC-Di_S) | 42400.1 | 42403.3 ± 0.4 |
| 2x(MCL-1:CC_Di_E1) | 42654.5 | 42657.2 ± 0.1 |
| 2x(MCL-1:CC-Di_E2) | 42740.5 | 42743.3 ± 0.2 |
| MCL-1:CC-Di_A | 21148.7 | 21149.9 ± 0.2 |
| MCL-1:CC-Di-B_S | 21197.2 | 21198.0 ± 0.3 |
| MCL-1:A:BS | 24611.0 | 24612.1 ± 0.1 |
| MCL-1(2):CC-Di-A:CC-Di-B_S | 49221.9 | Broad peak observed at 42400 Da ± 350 Da |
| 2x(MCL-1:CC-Di-A) | 42297.5 | |
| 2x(MCL-1:CC-Di-B_S) | 42394.5 | |
| MCL-1:CC-Tet_E1 | 21326.3 | 21327.4 ± 0.4 |
| 2x(MCL-1:CC-Tet_E1) | 42652.6 | 42655.8 ± 0.2 |

## Analytical ultracentrifugation

The sedimentation velocity experiments were performed on a Beckman Optima X-LA with an An-50-Ti rotor. The experiments were performed with 30 µM MCL-1 protein and peptide concentrations ranging from 30 µM to 120 µM depending on the ratio MCL-1 to peptide. A Tris buffer (50 mM Tris, 150 mM NaCl, pH 7.4) was used. The data was collected at 48-50 krpm at 5-min intervals for a total of 120 scans. SEDENTERP[4] was used to calculate buffer densities, viscosities and peptide partial specific volumes used in the fitting. The data was fitted to a continuous distribution using SEDFIT[5] at a 95% confidence level. The baseline, bottom of the cell, frictional coefficient ($f/f_0$), systematic time-invariant and radial-invariant noise were floated during fitting.

## Size exclusion chromatography

Size exclusion chromatography experiments of MCL-1 and peptide mixtures were performed on a superdex™ 75 increase 10/300 (Cytiva) column using a Jasco HPLC pump and Jasco UV detector. 30 µM MCL-1 was mixed with various concentrations of peptide ranging from 15 µM to 480 µM. Column and samples were buffered with Tris (20 mM Tris, 150 mM NaCl, pH 7.5). 20 µL of sample was injected and the chromatograms were recorded measuring UV absorbance at 230 nm at a flow rate of 0.8 mL/min. The data is shown as raw absorbance after subtraction of buffer absorbance.

### Peptide Synthesis

Peptides were prepared *via* standard solid phase peptide synthesis. All Fmoc-protected amino acids were purchased from Sigma-Aldrich or Novabiochem (Merck). Piperidine (Sigma-Aldrich), Oxyma (Ethyl(hydroxyamino)cyanoacetate) Novabiochem), 1,3-diisopropylcarbodiimide (DIC, Sigma Aldrich), trifluoroacetic acid (TFA, ThermoFisher), triisopropylsilane (TIPS, Sigma Aldrich), acetic anhydride (Sigma Aldrich), N,N-diisopropylethylamine (DiPEA, Acros Organics), 2,2'-(ethylenedioxy)diethanethiol (DoDT, Sigma Aldrich), 6-(Fmoc-amino)hexanoic acid (Fmoc-Ahx-OH, Sigma Aldrich), 5-carboxyfluorescein (FAM, Sigma Aldrich), acetonitrile (ACN, Sigma Aldrich), dimethylformamide (DMF, Fischer Scientific) were commercially available and used as is/upon arrival.

Peptides were prepared to be C-terminal amidated via Rink amide MBHA resin (0.8 mmol/g loading, Sigma Aldrich) and were synthesized with an automated microwave peptide synthesizer (Liberty Blue, CEM) at 0.05 mmol scale. Deprotection was performed with a 20% piperidine solution in DMF (5 mL repeated twice), followed by 5 DMF washes (5 mL), and coupling achieved with Oxyma/DIC with a ratio of resin:Fmoc-amino acid:Oxyma:DIC of 1:4.5:4.5:4.5. After the last coupling cycle a final deprotection step was performed to give a free N-terminus. For acetylated peptides, the resin-bound peptide was reacted with a 1:10:10 ratio of peptide:acetic anhydride:DiPEA in DMF (5 mL volume for 1 h); this step was performed twice, and the acetylated resin-bound peptide then washed twice with DMF (5 mL). For FAM-labelled peptides, the free N-terminus of the resin-bound peptide was coupled with Fmoc-Ahx-OH (ratio of resin:Fmoc-Ahx-OH:Oxyma:DIC as 1:4.5:4.5:4.5), washed 3 times with DMF and Fmoc removed with 20% piperidine/DMF (5 mL, repeated twice) followed by 3 DMF washes (5 mL volume); FAM was coupled to the resin-bound peptide with a 1:3:4.5:4.5 ratio of resin:FAM:Oxyma:DIC and the resin then washed twice with DMF (5 mL).

A cleavage cocktail (TFA:water:TIPS:DoDT 92.5:2.5:2.5:2.5 by volume, 5 mL, 2 h) was used to simultaneously remove side chain protecting groups and cleave the peptide from the resin. The resin then was removed from the mixture by filtration and the cleavage cocktail reduced under a flow of nitrogen gas, triturated with cold diethyl ether, the mixture centrifuged (10 min, 6,000 rpm, repeated twice), and the diethyl ether decanted to give the crude peptide as a pellet. Crude peptides were purified via RP-HPLC on an Agilent 1260 Infinity system (containing a diode array UV detector) with a Kinetex EVO C18 column (250 mm x 21.2 mm, Phenomenex, 12 mL/min flow rate) column using a linear gradient of 30% acetonitrile/water to 95% acetonitrile/water. Fractions containing the pure peptide were confirmed by HPLC-MS, pooled, frozen and lyophilized to give isolated peptide for further analysis. Analytical purity was assessed on an Agilent 1260 Inifinity HPLC system equipped with an Ascentis Express C18 column (250 mm x 4.6 mm, 5 um, Supelco, 1.5 mL/min flow rate). High resolution mass spectra were recorded with a BrukerDaltonics maXis Impact using electrospray ionization (ESI), or a Waters Synapt GS-2 using ESI. Before use in experiments, concentrations were calculated using the absorbance at A280 on a NanoDrop One (Thermo Scientific) if the peptide contained an aromatic amino acid; if the peptide had no aromatic amino acid stock concentrations were calculated by mass.

**Sequences for the NOXA-B, $BID_{wt}$, and FAM-Ahx-$BID_{wt}$ peptides.**

| Peptide | Sequence |
|---|---|
| NOXA-B | Ac-AAQLRRIGDKVNLRQKLLN-$NH_2$ |
| $BID_{wt}$ | Ac-EDIIRNIARHLAQVGDS[Nle]DRSIW-$NH_2$ |
| FAM-Ahx-$BID_{wt}$ | FAM-Ahx-EDIIRNIARHLAQVGDS[Nle]DRSIW-$NH_2$ |

### Analytical HPLC and MS of peptides

Agilent 1260 Inifinity HPLC system equipped with an Ascentis Express C18 column (250 mm x 4.6 mm, 5 um, Supelco, 1.5 mL/min flow rate) and BrukerDaltonics maXis Impact using electrospray ionization (ESI), or a Waters Synapt GS-2 using ESI.

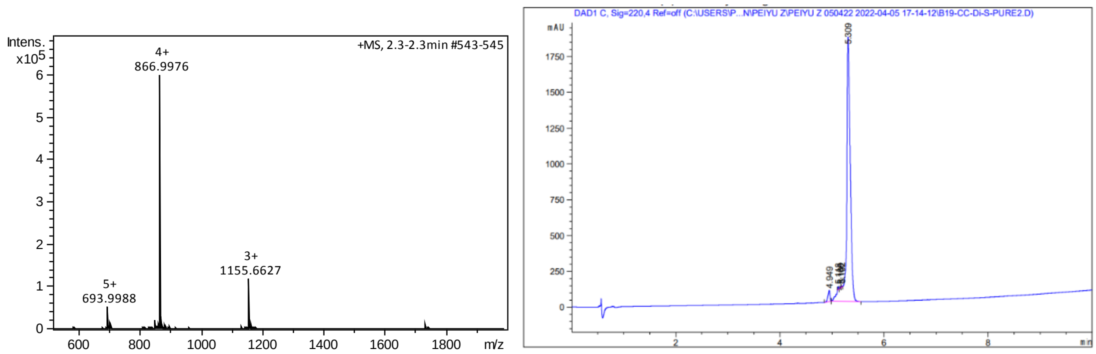


**Mass spectrometry and analytical HPLC for CC-Di_S.** Predicted MW 3465.032. Observed MW 3464.944.

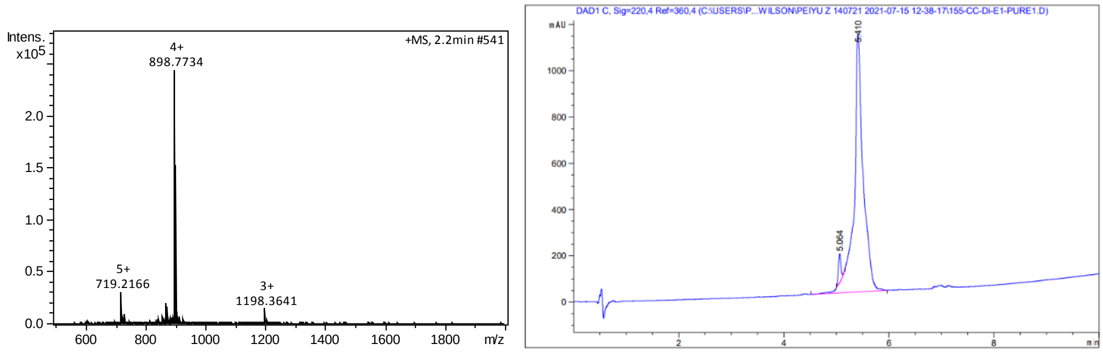


**Mass spectrometry and analytical HPLC for CC-Di_E1.** Predicted MW 3592.218. Observed MW 3592.062.

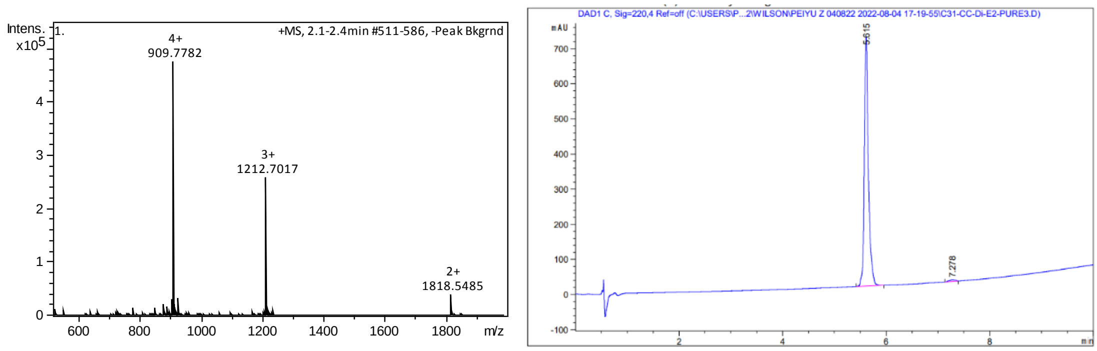


**Mass spectrometry and analytical HPLC for CC-Di_E2.** Predicted MW 3635.243. Observed MW 3635.077.

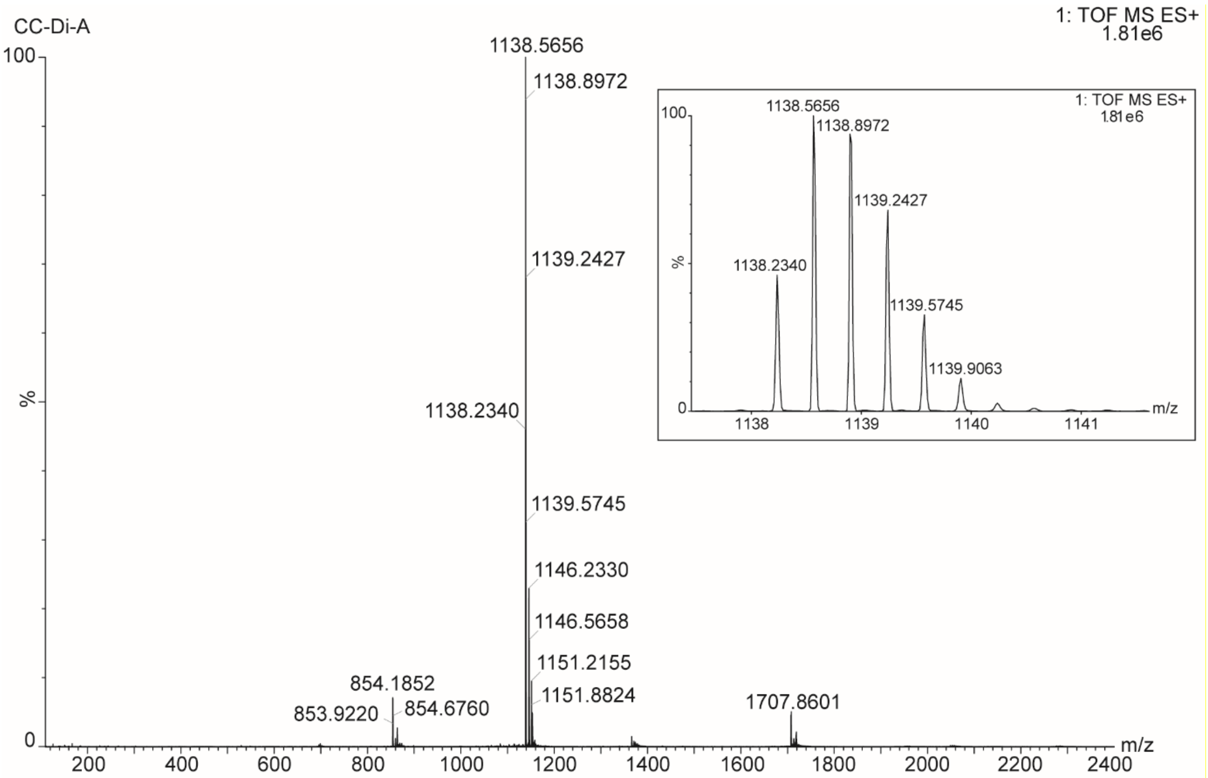


**Mass spectrometry data for CC-Di-A.** Predicted 3,411.694. Observed 3,411.462.

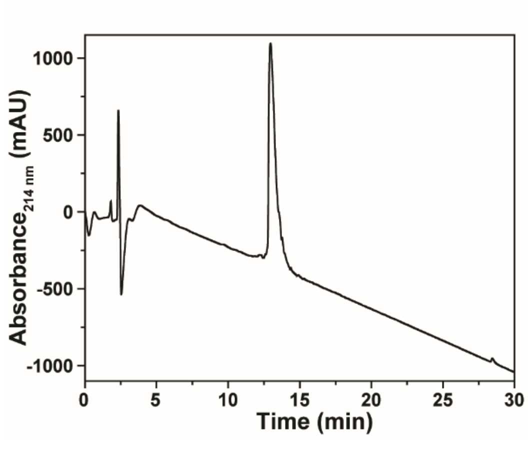


**Analytical HPLC for CC-Di-A.**

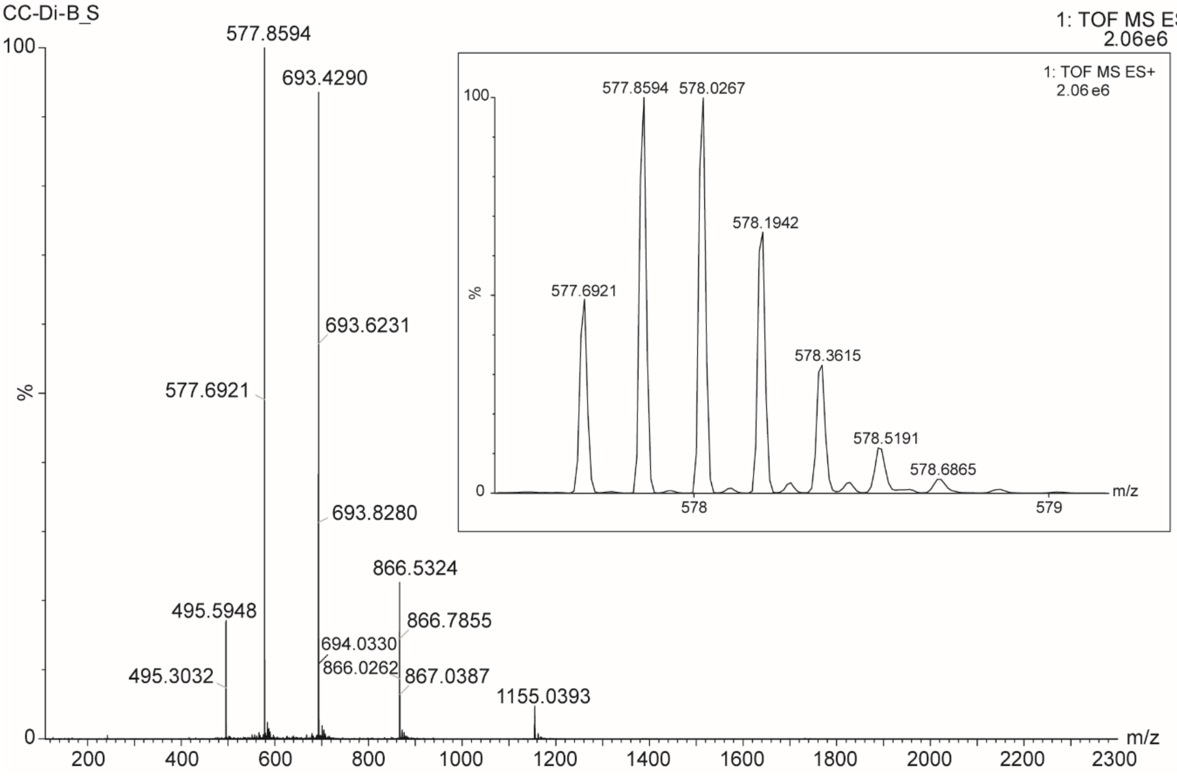


**Mass spectrometry data for CC-Di-B_S.** Predicted 3,460.103. Observed 3,459.673.

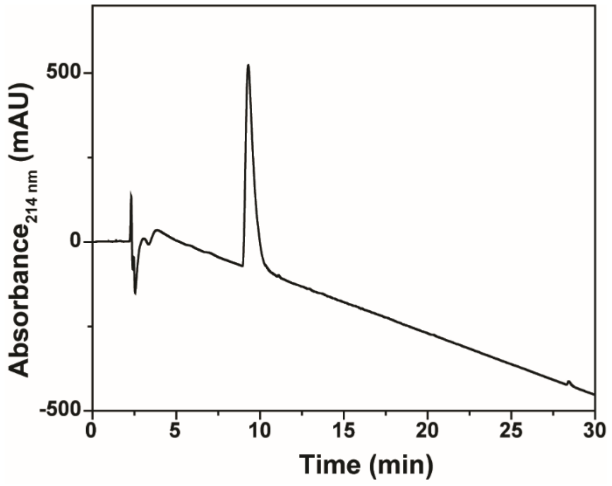


**Analytical HPLC for CC-Di-B_S.**

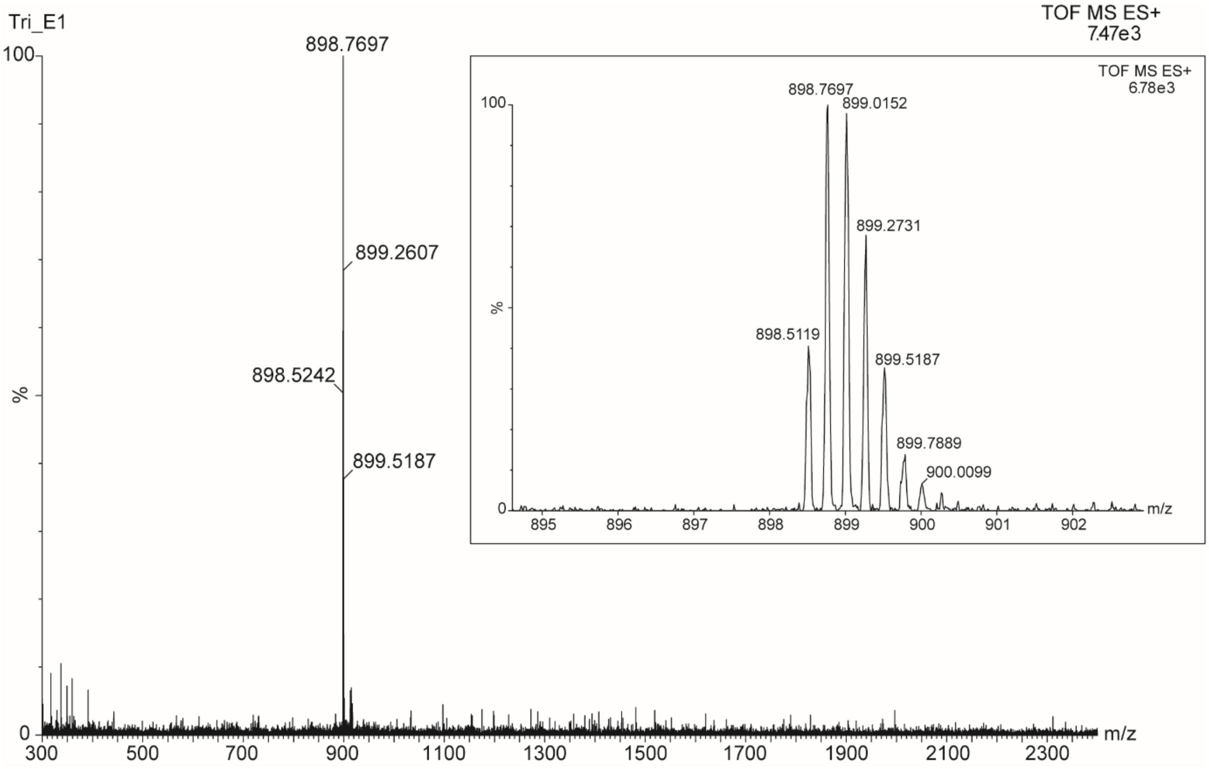


**Mass spectrometry data for CC-Tri_E1.** Predicted 3,590.046. Observed 3,589.728.

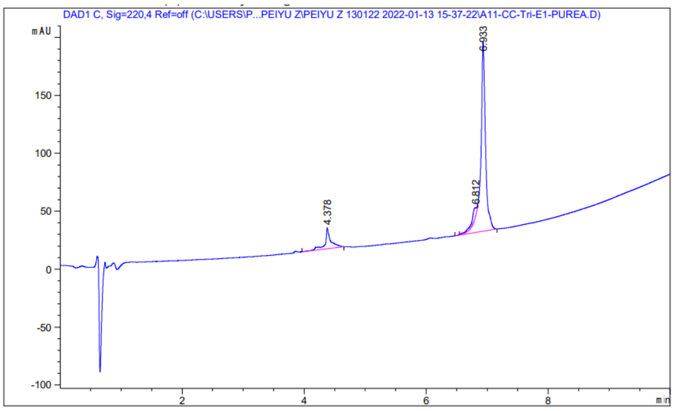


**Analytical HPLC for CC-Tri_E1.**

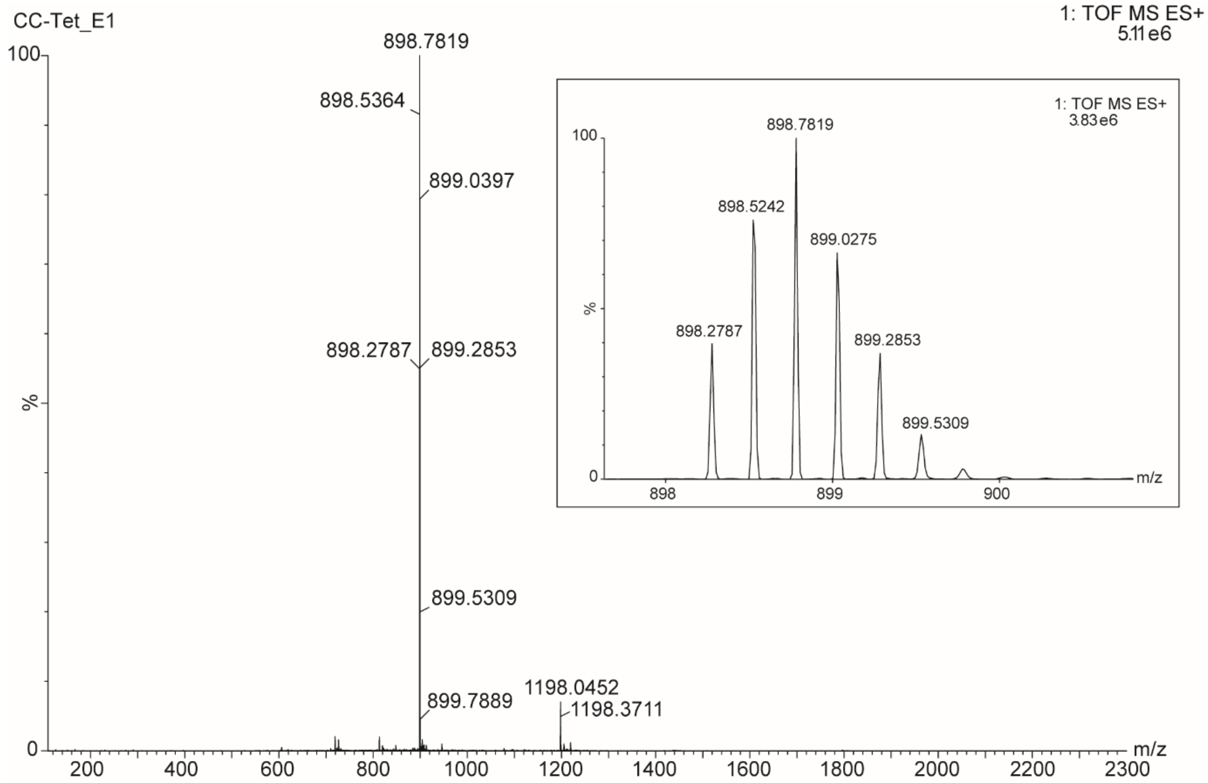


**Mass spectrometry data for CC-Tet_E1.** Predicted 3,589.087. Observed 3,588.795.

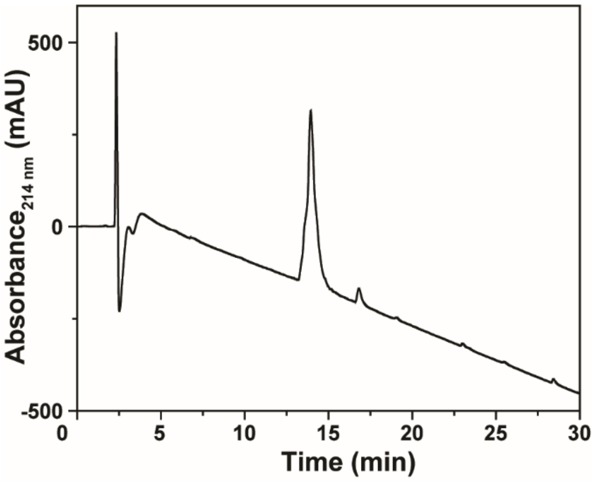


**Analytical HPLC for CC-Tet_E1.**

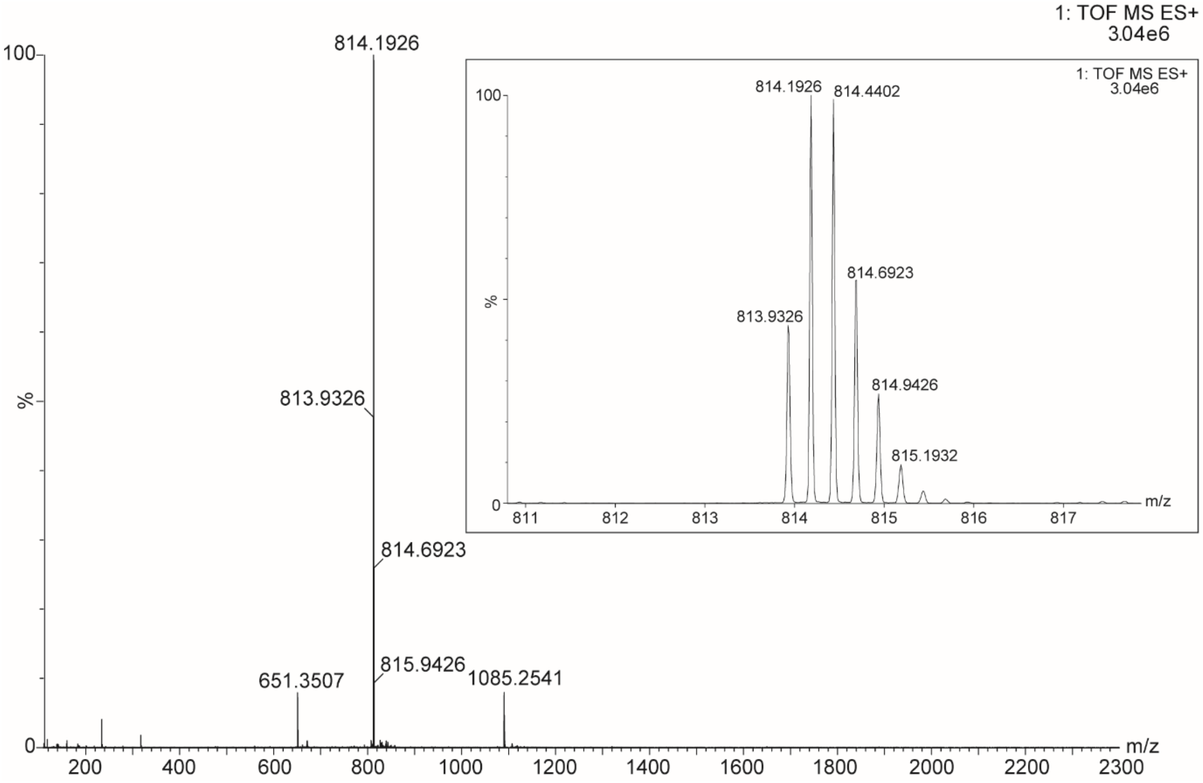


**Mass spectrometry data for Mono_E1.** Predicted 3,252.711. Observed MW 3,251.690.

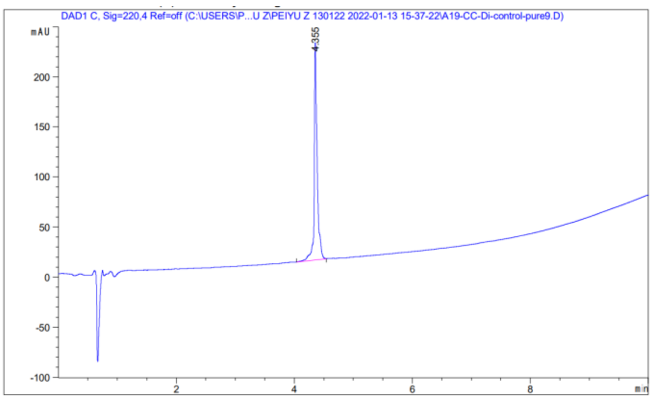


**Analytical HPLC for Mono_E1.**

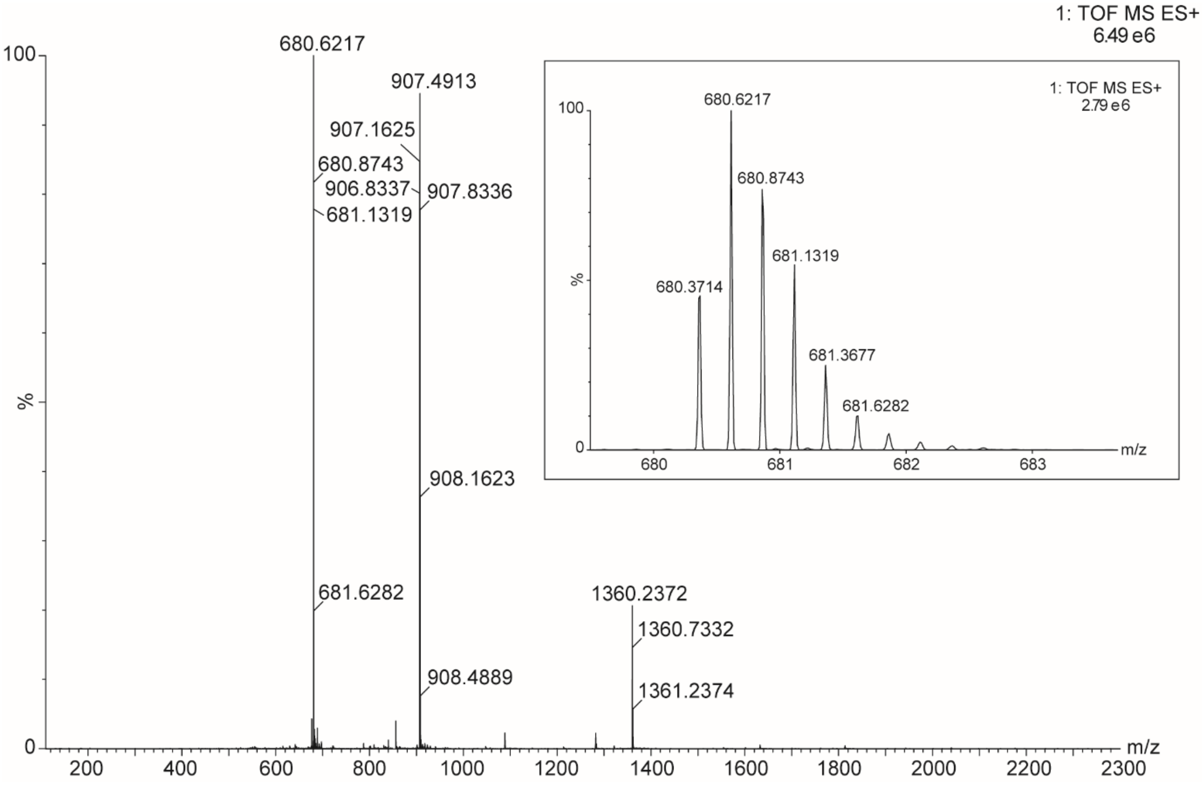


**Mass spectrometry data for $BID_{wt}$.** Predicted 2,717.441. Observed 2,717.446.

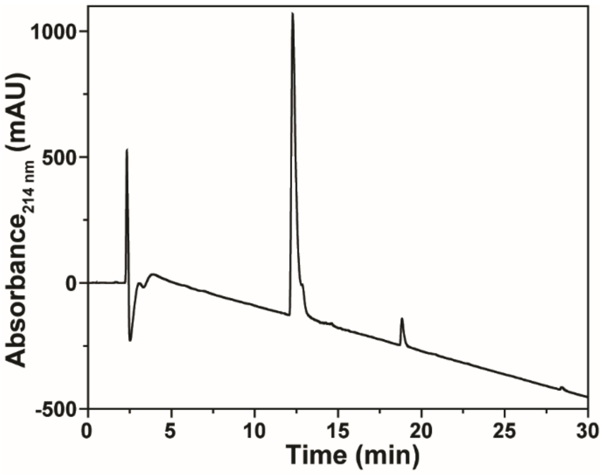


**Analytical HPLC for $BID_{wt}$.**

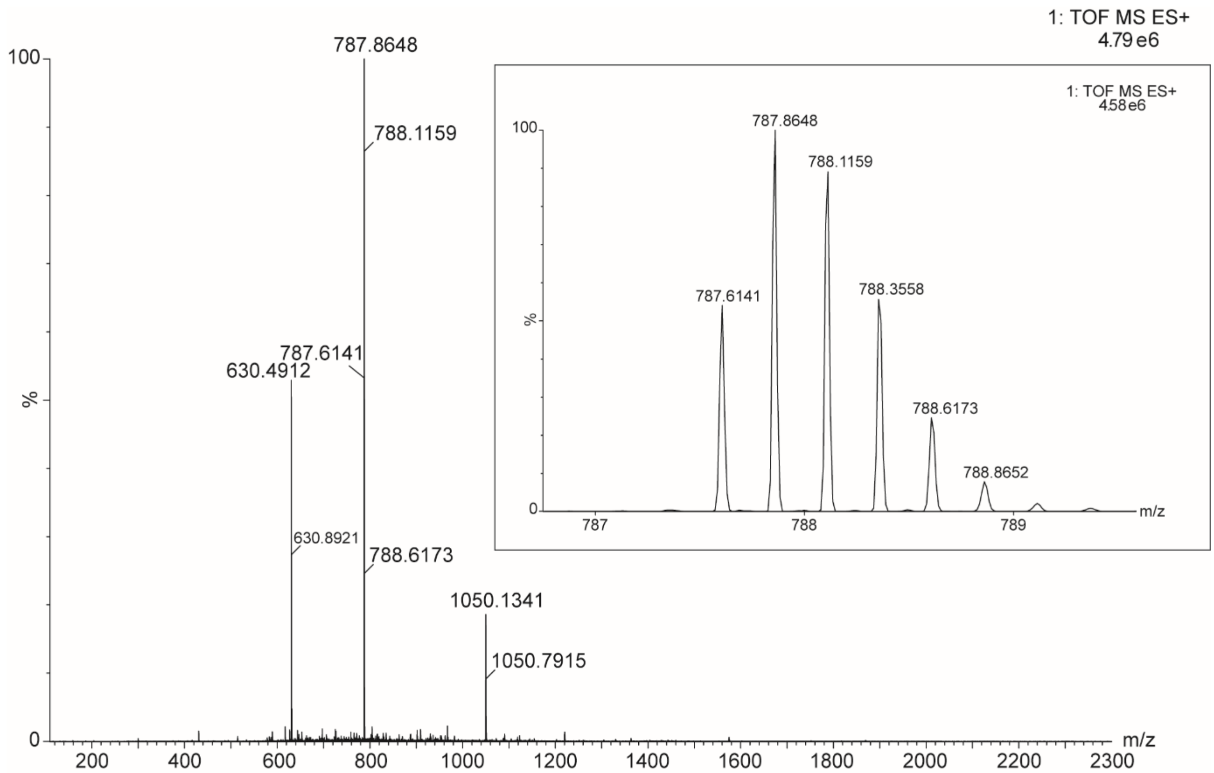


**Mass spectrometry data for FAM-Ahx-BID$_{wt}$.** Predicted 3,146.563. Observed 3,146.416.

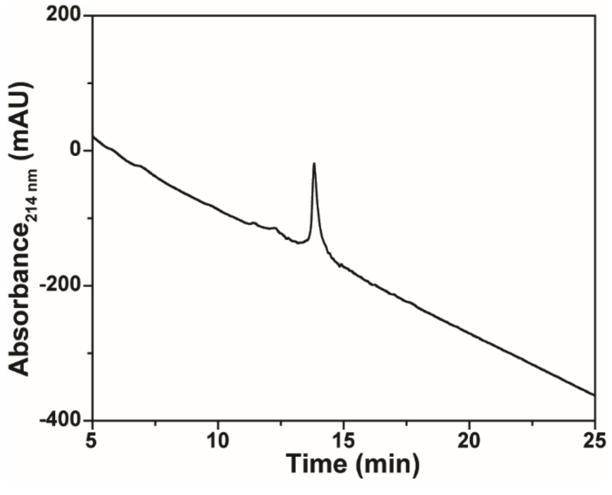


**Analytical HPLC for FAM-Ahx-BID$_{wt}$.**

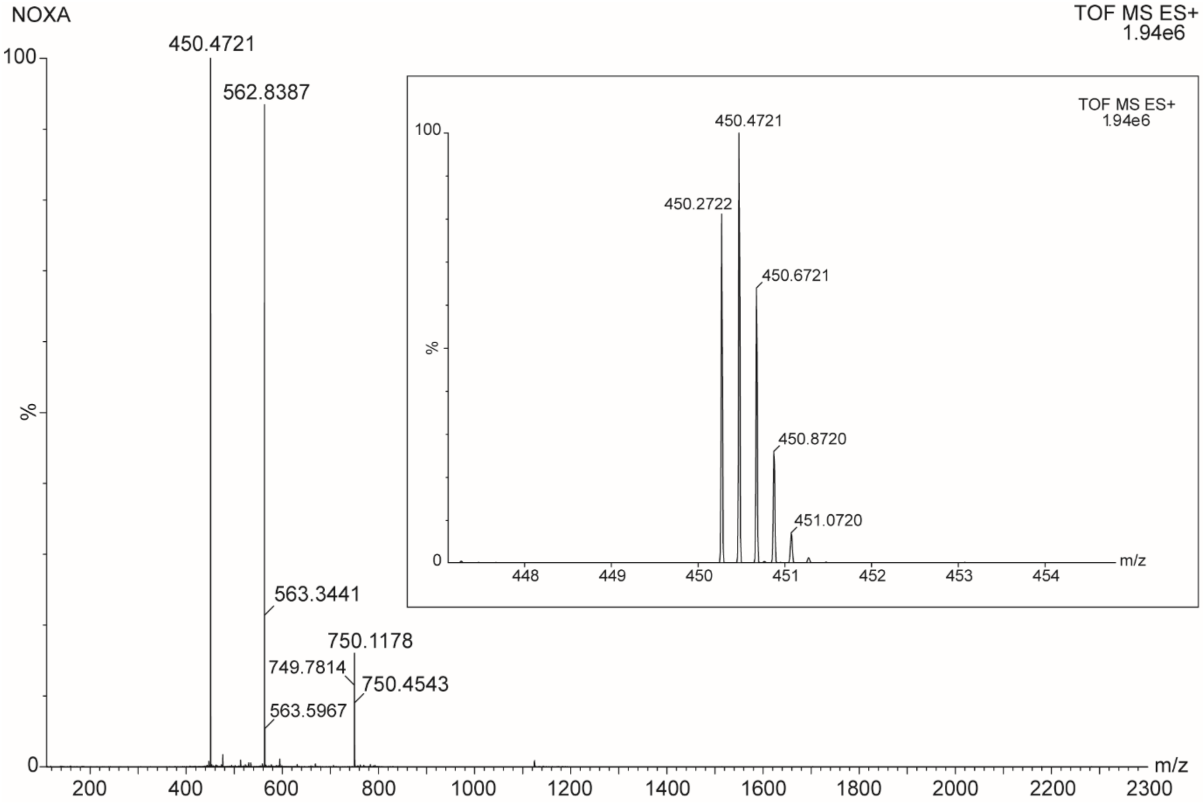


**Mass spectrometry data for NOXA-B.** Predicted 2,246.334. Observed 2,245.961.

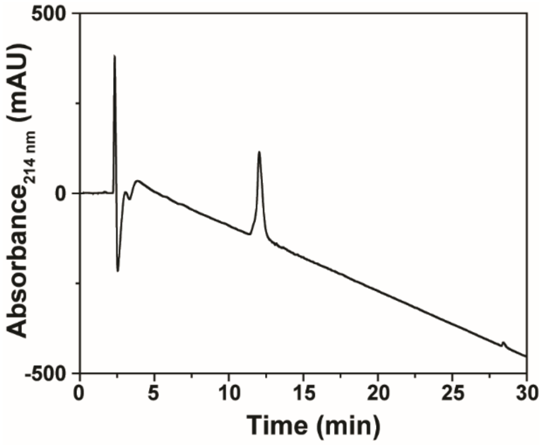


**Analytical HPLC for NOXA-B.**

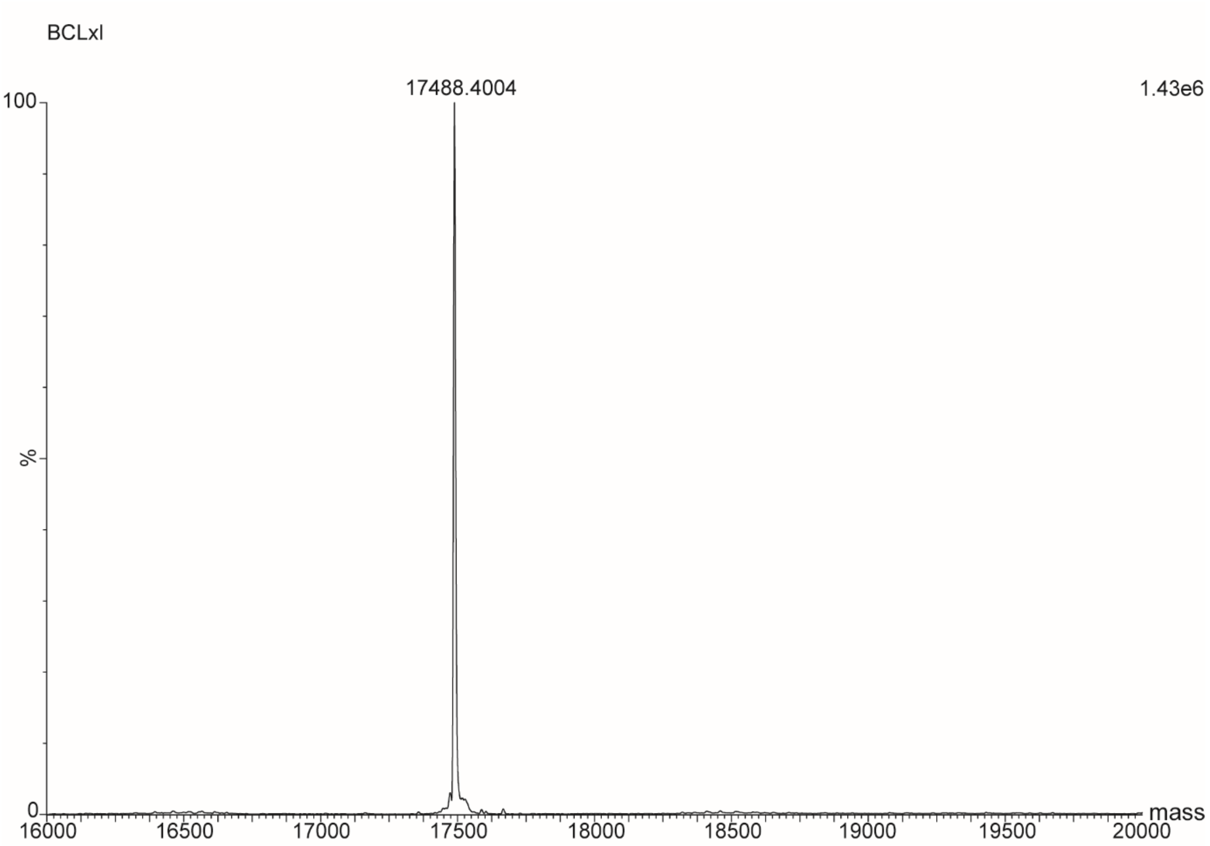


**Deconvoluted mass spectrometry data for BCL-$_{xL}$.** Predicted 17,489.5. Observed 17488.4.

**References**


1. Mirdita, M.; Schutze, K.; Moriwaki, Y.; Heo, L.; Ovchinnikov, S.; Steinegger, M., ColabFold: making protein folding accessible to all. *Nat Methods* **2022,** *19* (6), 679-682.
2. Jumper, J.; Evans, R.; Pritzel, A.; Green, T.; Figurnov, M.; Ronneberger, O.; Tunyasuvunakool, K.; Bates, R.; Zidek, A.; Potapenko, A.; Bridgland, A.; Meyer, C.; Kohl, S. A. A.; Ballard, A. J.; Cowie, A.; Romera-Paredes, B.; Nikolov, S.; Jain, R.; Adler, J.; Back, T.; Petersen, S.; Reiman, D.; Clancy, E.; Zielinski, M.; Steinegger, M.; Pacholska, M.; Berghammer, T.; Bodenstein, S.; Silver, D.; Vinyals, O.; Senior, A. W.; Kavukcuoglu, K.; Kohli, P.; Hassabis, D., Highly accurate protein structure prediction with AlphaFold. *Nature* **2021,** *596* (7873), 583-589.
3. Bryant, P.; Pozzati, G.; Zhu, W.; Shenoy, A.; Kundrotas, P.; Elofsson, A., Predicting the structure of large protein complexes using AlphaFold and Monte Carlo tree search. *Nat Commun* **2022,** *13* (1), 6028.
4. Philo, J. S., SEDNTERP: a calculation and database utility to aid interpretation of analytical ultracentrifugation and light scattering data. *Eur. Biophys. J.* **2023,** *52* (4), 233-266.
5. Schuck, P.; Perugini, M. A.; Gonzales, N. R.; Howlett, G. J.; Schubert, D., Size-Distribution Analysis of Proteins by Analytical Ultracentrifugation: Strategies and Application to Model Systems. *Biophys. J.* **2002,** *82* (2), 1096-1111.
